# Plasma microRNA signatures of aging and their links to health outcomes and mortality: findings from a population-based cohort study

**DOI:** 10.1186/s13073-025-01437-5

**Published:** 2025-06-25

**Authors:** Lieke M. Kuiper, Michelle M. J. Mens, Julia W. Wu, Jaap Goudsmit, Yuan Ma, Liming Liang, Albert Hofman, Trudy Voortman, M. Arfan Ikram, Jeroen G. J. van Rooij, Joyce B. J. van Meurs, Mohsen Ghanbari

**Affiliations:** 1 Department of Internal Medicine, Erasmus MC University Medical Center, Rotterdam, The Netherlands; 2 Center for Prevention, Lifestyle and Health, National Institute for Public Health and Environment (RIVM), Bilthoven, the Netherlands; 3 Department of Epidemiology, Erasmus MC University Medical Center, Rotterdam, The Netherlands; 4 Department of Social and Behavioral Sciences, Harvard T.H. Chan School of Public Health, Boston, MA USA; 5 Department of Epidemiology, Harvard T.H. Chan School of Public Health, Boston, MA USA; 6 Department of Immunology & Infectious Diseases, Harvard T. H. Chan School of Public Health, Boston, MA USA; 7 Department of Biostatistics, Harvard T.H. Chan School of Public Health, Boston, MA USA; 8 Meta-Research Innovation Center at Stanford (METRICS), Stanford University, Stanford, CA USA; 9 Department of Orthopaedics & Sports Medicine, Erasmus MC University Medical Center, Rotterdam, the Netherlands

**Keywords:** MicroRNA, Aging, Biomarker, Biological age, Mortality, Frailty

## Abstract

**Background:**

MicroRNAs are small non-coding RNAs that regulate gene expression post-transcriptionally and show differential expression in various tissues with aging phenotypes. Detectable in circulation, extracellular microRNAs reflect (patho)physiological processes and hold promise as biomarkers for healthy aging and age-related diseases. This study aimed to explore plasma extracellular microRNAs as a biological aging indicator and their associations with health outcomes using population-level data.

**Methods:**

We quantified plasma expression levels of 2083 extracellular microRNAs using targeted RNA-sequencing in 2684 participants from the population-based Rotterdam Study cohort. The training and test sets included 1930 participants from the advanced-aged initial and second subcohort (RS-I/RS-II; median age: 70.6), while the validation set comprised 754 participants from the middle-aged fourth subcohort (RS-IV; median age: 53.5). Based on 591 microRNAs well-expressed in plasma, we examined differential expression of microRNAs with chronological age, PhenoAge—a composite score of age and nine multi-system blood biomarkers—the frailty index, and mortality. Next, elastic net models were employed to construct composite microRNA-based aging biomarkers predicting chronological age (mirAge), PhenoAge (mirPA), frailty index (mirFI), and mortality (mirMort). The association of these aging biomarkers with different age-related health outcomes was assessed using Cox Proportional Hazard, linear regression, and logistic regression models in the test and validation sets.

**Results:**

We identified 188 microRNAs differentially expressed with chronological age within the RS-I/RS-II advanced-aged population (*n*_training_ = 1158, *n*_test_ = 772), of which 177 microRNAs (94.1%) were replicated in the middle-aged RS-IV subcohort (*n*_validation_ = 754). Moreover, 227 miRNAs showed robust associations with PhenoAge, 61 with FI, and 16 with 10-year mortality independent of chronological age. Subsequently, we constructed four plasma microRNA-based aging biomarkers: mirAge with 108, mirPA with 153, mirFI with 81, and mirMort with 50 miRNAs. Elevated scores on these microRNA-based aging biomarkers were associated with unfavorable health outcomes, including lower subjective physical functioning and self-reported health and increased mortality and frailty risk, but not with first- or multi-morbidity. Overall, larger effect estimates were observed for mirPA, mirFI, and mirMort compared to mirAge.

**Conclusions:**

This study describes distinct plasma microRNA-aging signatures and introduces four microRNA-based aging biomarkers with the potential to identify accelerated aging and age-related decline, providing insights into the intricate process of human aging.

**Supplementary Information:**

The online version contains supplementary material available at 10.1186/s13073-025-01437-5.

## Background

The susceptibility to most common diseases increases with age [1]. However, chronological age does not account for the heterogeneity in age-related disease susceptibility among individuals, underscoring the need for biomarkers of biological age to assess individual health status [2–4]. In recent years, aging biomarkers using molecular information such as epigenetic [4], transcriptomic [5], and metabolomic [6, 7] information have been developed. Earlier research showed that first-generation aging biomarkers, trained solely on chronological age, were outperformed by second-generation aging biomarkers that incorporated information on health status or mortality in reflection of frailty and prediction of mortality [8–13].

MicroRNAs (miRNAs) are small non-coding RNAs that regulate gene expression post-transcriptionally [14, 15]. Previous studies showed aging-related miRNA expression in various tissues [16–24]. Furthermore, a composite qPCR miRNA-based aging biomarker on almost 150 whole-blood miRNAs was associated with all-cause mortality and age-related traits in the Framingham Heart Study [23]. However, despite the standard plasma storage in large biobank cohorts, no plasma cell-free miRNA-based aging biomarkers have been developed at the population level. Circulating cell-free miRNAs, released from cells into biological fluids, are remarkably stable in extracellular environments and considered to reflect active processes in the body, making them potential biomarkers for non-invasive personalized molecular diagnosis [25]. In a previous study, we demonstrated plasma circulating miRNAs that were differentially expressed with chronological age and PhenoAge—a composite score of chronological age and nine multi-system blood biomarkers—using a novel RNA sequencing approach on over 2000 extracellular miRNAs [24].

In the current study, we aimed to build a miRNA biomarker for biological aging, also referred from plasma-based extracellular miRNA levels. Using elastic net regression, we built four miRNA biomarkers of biological age trained on chronological age, PhenoAge, the frailty index, and mortality. We evaluated their performance in the prediction of (un)healthy aging by assessing their association with all-cause mortality and different health outcomes.

## Methods

### Study population

This study was conducted using data from the Rotterdam Study (RS). The design of the RS cohort has previously been described elsewhere [26]. Briefly, RS is a large prospective, population-based cohort study conducted among middle-aged and older people in the suburb Ommoord in Rotterdam, the Netherlands. The RS has four subcohorts, and participants (> 40) are followed every 3–5 years. The initial cohort (RS-I) started out in 1990 with 7983 participants aged 55 years and over. In 2000, the RS was extended with a second subcohort (RS-II) of 3011 participants who moved to Ommoord or turned 55 years old. The third subcohort (RS-III) started in 2006 with 3932 inhabitants aged 45 years and over. The most recent extension took place in 2016 and led to the inclusion of 3005 individuals aged 40 and older, forming the fourth subcohort (RS-IV). The RS has been approved by the Medical Ethics Committee of the Erasmus MC and by the review board of the Dutch Ministry of Health, Welfare, and Sports (1,068,889–159,521-PG).

**Fig. 1:**
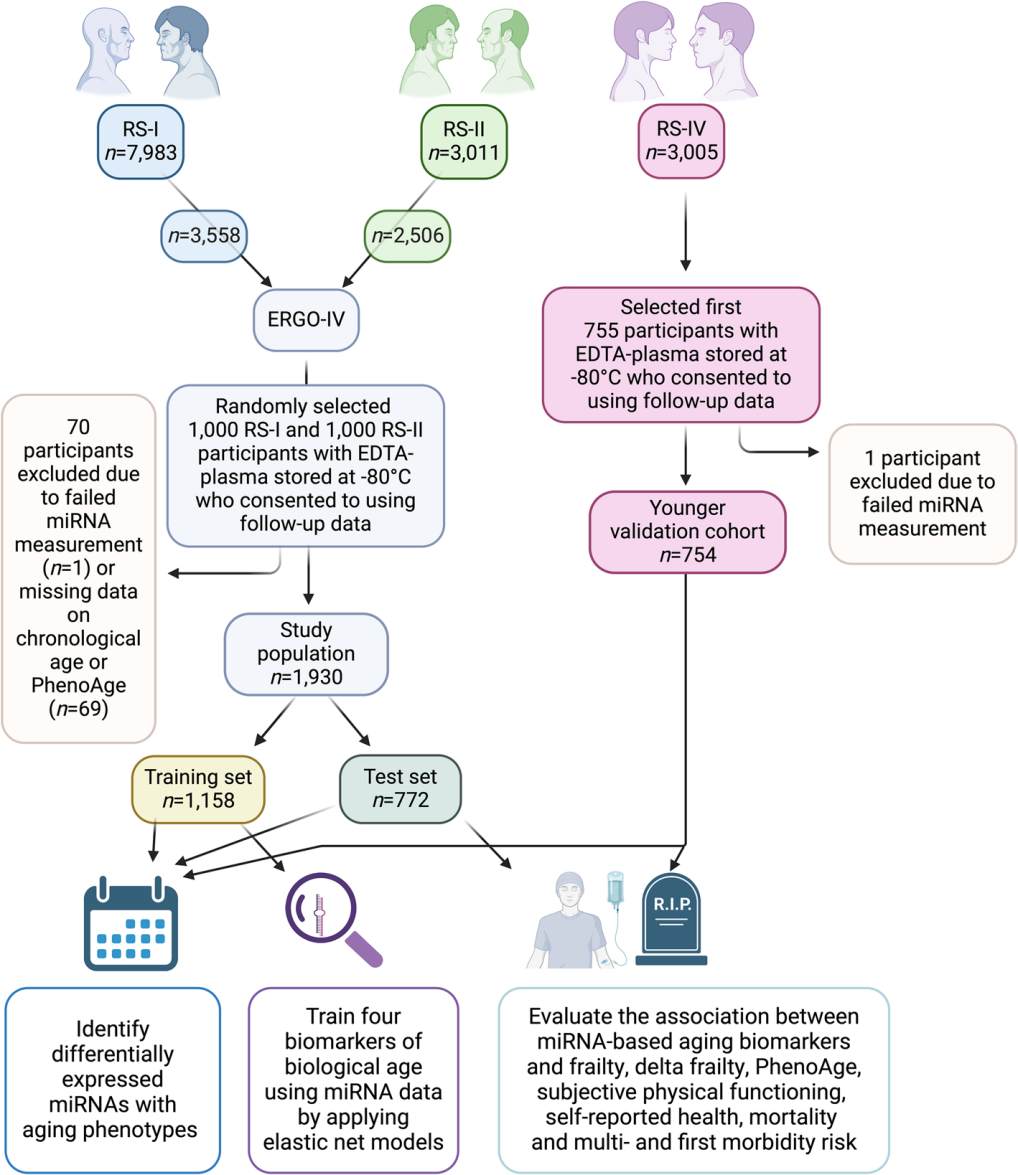
Study flow chart. Illustration of the study population selection: 2000 individuals were randomly selected from the fourth round of Rotterdam Study-I (RS-I-4) and second round of Rotterdam Study-II (RS-II-2) with blood samples collected between 2002 and 2005. Data analyzed in this study included 1930 participants with physiological, clinical, and miRNA assay information available. The miRNA data of 754 participants from the RS-IV were also used for validation and sensitivity analyses

Plasma miRNA levels were measured in 2755 participants randomly selected from three RS subcohorts: 1000 from the fourth visit of the initial subcohort (RS-I-4), 1000 from the second visit of the second subcohort (RS-II-2), and 755 from the first visit of the fourth subcohort (RS-IV-1). The RS-I-4 and RS-II-2 visits were conducted between 2002 and 2005, with participants invited for re-examination approximately every 5 years, while the younger RS-IV-1 cohort was examined between 2016 and 2020. For RS-I and RS-II, participants with missing miRNA profiles or incomplete data on chronological age or PhenoAge were excluded to enhance a proper comparison to our previous pipeline [24]. This resulted in the removal of 70 participants, leaving 1930 for analysis (Fig. 1). In RS-IV, only one participant was excluded due to a failed miRNA measurement, resulting in 754 participants, which served as the validation cohort (Fig. 1).

### Blood collection and storage

Fasting (8–14 h) blood samples of RS participants were obtained during each visit to the research center. EDTA plasma and serum, collected in Vacutainer tubes (Becton Dickinson), were processed within 60 minutes using a standardized protocol and stored at −80°C. Blood cell counts (EDTA Vacutainer tubes, Becton Dickinson) were measured using the XS800i Hematology Analyzer (Sysmex). These measurements included absolute counts of erythrocytes, granulocytes, lymphocytes, and platelets.

### MiRNA expression profiling

Plasma miRNA levels were determined using the HTG EdgeSeq miRNA Whole Transcriptome Assay (WTA), which is a next-generation sequencing (NGS) application that measures the expression of 2083 human miRNAs. HTG EdgeSeq platform works as a targeted probe library preparation, wherein probes are attached to their intended targets. The assay is ideal for the characterization of miRNA expression patterns and also measures the expression of 13 housekeeping genes as control, allowing for greater flexibility in data normalization and analysis. A total volume of 50 μL of plasma, for two re-measurements that generally is sufficient to obtain a valid result for all samples, was sent to the HTG Molecular Diagnostics, Inc. (AZ, USA) for sequencing by the Illumina NextSeq 500 sequencer (Illumina, San Diego, CA, USA). Quantification of miRNA expression data was provided as data tables of raw, quality control (QC) raw, counts per million (CPM), and median normalized counts. Log2 CPM standardization was used to transformed counts and adjusted for total reads within a sample. MiRNAs with Log2 CPM < 1.0 were indicated as not expressed in the samples. Out of the 2083 profiled miRNAs, 591 miRNAs were expressed at good levels in plasma, which are those with > 50% values above Lower Limit of Quantification (LLOQ). The LLOQ level is based on a monotonic decreasing spline curve fit between the expression means and standard deviations of all miRNAs.

### Ascertainment of outcomes

Chronological age was determined as the time in years between birth and blood sampling. PhenoAge, a composite score of chronological age and nine multi-system blood biomarkers (albumin, creatinine, glucose, [log] C-reactive protein, lymphocyte percent, mean cell volume, red blood cell distribution width, alkaline phosphatase, and white blood cell count), was directly applied to the Rotterdam Study data, as previously described in detail [24]. Data on PhenoAge was not available in RS-IV. We calculated the frailty index (FI), a measure of accumulated age-related deficits designed in the Rotterdam Study by Schoufour et al. [27], including deficits that were available in RS-I-4 and RS-II-2. The 38 deficits included in the adapted FI can be found in Additional file 1. The FI was calculated, if a person had information on at least 20 deficits before multi-chain imputation, as $\frac{\text{the number of deficits}}{38}$. Additionally, we calculated delta FI by subtracting the FI at RS-I-4 or RS-II-2 from the corresponding value at the subsequent visit, approximately 5 years later. Frailty information was not available in RS-IV.

All-cause mortality was defined as participants who died from any cause during the total follow-up period, which concluded on November 23rd, 2023. Ten-year mortality was defined as dying within 10 years after blood sampling. Information on participants’ vital status was obtained biweekly via municipal population registries and general practitioners’ and hospitals’ databases.

In addition, secondary outcomes were included in the study. Self-reported health was based on the answer to the question: “How would you rate your health compared to your peers?” and could be worse, the same, or better. Information on self-reported health was only available in RS-IV. Overall subjective physical functioning was assessed by two scores: the basic activities of daily living (BADL) and the instrumental activities of daily living (IADL) [28]. BADL was determined by the Dutch version of the disability index from the Stanford Health Assessment Questionnaire, including 20 items across eight components (dressing, arising, eating, walking, hygiene, grip, reach, and activities) [29]. Each item, scored from 0 to 3, reflects varying degrees of ability (0 = no difficulty; 1 = some difficulty; 2 = much difficulty; 3 = unable to), with the component score being the highest item value. The BADL, ranging from 0 to 24, sums all component scores. IADL was measured using the IADL scale comprising eight tasks requiring cognitive ability for self-reliant living: telephone use, medication, shopping, travel, finances, laundry, housekeeping, and preparing meals, again scored 0–3 [30]. For adapted phone use, a score of 2 represented much difficulty. IADL was calculated as the sum of the components ranging from 0 to 24.

First morbidity was defined as the moment of diagnosis of either diabetes mellitus type 2, stroke, coronary heart disease (CHD), dementia, chronic obstructive pulmonary disease (COPD), or cancer after exclusion of prevalent cases. Multi-morbidity was defined as the moment of the second diagnosis of one of these major morbidities after the exclusion of participants who were already diagnosed with two of these morbidities. Ascertainment of the different morbidities can be found in Additional file 2. Follow-up was truncated on January 1st, 2015.

### Ascertainment of covariates and demographic factors

A questionnaire at baseline provided information on the sex of participants. In our analyses, we adjusted for the RS-subcohort to mitigate any potential cohort-specific differences. Furthermore, we incorporated measured red cell counts, along with the white blood count percentage of lymphocytes and monocytes, to adjust for miRNAs originating mainly from these cells. To address technical bias, we included plate number and inner/outer well as covariates. The classification of the outer well was assigned to samples located in rows A, G, or H or positioned in columns 1, 11, or 12. When chronological age was not the outcome of interest, it was included as a covariate. When delta FI was the outcome of interest, analyses were adjusted for baseline FI. Genetic ancestry was determined per RS-subcohort for all participants with genetic data, not limited to those in this study. Cleaned genotypes were merged with HapMap CEU (build 36) [31], pruned for linkage equilibrium, and analyzed using ADMIXTURE (using default settings) [32]. Cross-validation for 1 to 8 HapMap ancestral populations identified groups with ≥ 50% genetic material from one ancestry. For participants without genetic data, ancestry was assigned if ≥ 3 grandparents were born in the same region.

### Differentially expressed miRNAs in relation to aging phenotypes

We performed a single random 60:40 data split between the batch of participants from the RS-I and RS-II cohorts to generate distinct training and test datasets for the purpose of cross-validation. We determined the difference in baseline characteristics between the training and test set and between the training and younger validation set using a Student’s *t*-test for continuous variables and *χ*^2^ test for binary variables. To identify chronological age-, PhenoAge-, FI-, and 10-year mortality-related miRNA expression in plasma, we used DESeq2 [33] to analyze the miRNA count data. For each analysis, we adjusted for chronological age (except when it was the primary outcome), sex, cell counts, RNA sequencing plate, inner/outer well position, and RS-subcohort in the training and test sets. We applied Benjamini and Hochberg false discovery rate (FDR)-correction to control for multiple testing [34]. We cross-validated the FDR-significant associations in the test set, adjusting for the same covariates. We determined robust associations as associations that remained FDR-significant in the test set. Only in the case of chronological age, we were able to validate the robust associations in the separate younger validation set (RS-IV). We constructed four volcano plots (with –log10(FDR-corrected *p*-value) on the *y*-axis and fold change of mean expression level on the *x*-axis) in the training set to display the significance and magnitude of each bivariate association. Using the R-package *ComplexUpset* we created a plot displaying the overlap of miRNAs univariately associated with each of the outcomes.

We performed sensitivity analyses in the training set to determine the influence of the selection of only well-expressed miRNAs for our analyses by varying the cut-off value for defining well-expressed miRNAs from 50 to 25% and 0% expression values.

### Tissue-specific expression and pathway analysis

We assessed miRNA tissue specificity using the Tissue Specificity Index (TSI) from miRNATissueAtlas2 [35], an online repository comprising 188 biological samples from six individuals (two women and four men). The TSI assigns miRNAs a value from 0 to 1, where 1 indicates expression restricted to a single organ or organ system and 0 indicates expression found in all organ or organ systems and was available for 575 of the 591 well-expressed miRNAs. We classified miRNAs with a TSI > 0.8 as tissue-specific, while those with a TSI ≤ 0.8 were considered multi-tissue miRNAs. Fisher’s exact test was then applied to evaluate whether miRNAs differentially expressed with chronological age, PhenoAge, the frailty index, or ten-year mortality were over- or underrepresented in specific tissues or among non-specific miRNAs.

Using the online database miRDB v6.0 [36], we identified human target genes for our miRNAs, applying an 80% confidence threshold to define target genes. Of the 591 well-expressed miRNAs, 571 could be matched to at least one target gene. For the differentially expressed miRNAs associated with chronological age, 12 could not be matched to any target gene. Similarly, 11 miRNAs associated with PhenoAge, 2 with FI, and 1 miRNA associated with ten-year mortality lacked target gene matches. All unmatched miRNAs were excluded from the subsequent overrepresentation analyses. We then used the *org.Hs.eg.db* R-package to convert the predicted gene symbols to ENTREZ IDs. Overrepresentation analyses of biological process Gene Ontology (GO) terms and KEGG pathways were subsequently conducted on the target genes of the differentially expressed miRNAs using the clusterProfiler R-package. Given that miRNAs target multiple genes and individual genes may be targeted by multiple miRNAs, all predicted genes, including duplicates, were used as the background. The analyses were FDR-adjusted, with significance defined as an adjusted *p*-value < 0.05 and a *Q*-value < 0.2. Finally, the results were visualized using the *enrichplot* R-package.

### MiRNA biological age prediction

We applied the *edgeR* R-package [37] to perform trimmed mean of M-values normalization separately for the count values of the 591 miRNAs in the RS-I and RS-II cohorts (*n* = 1930) and the RS-IV cohort (*n* = 754). This normalization process was used to scale the data between samples. We then recalculated the log2(CPM) values. We conducted four elastic net regression models in the training set (*n* = 1158) with the following outcomes: (1) chronological age, (2) PhenoAge, (3) FI, and (4) all-cause mortality using the *glmnet* R-package [38, 39]. The elastic net modeling method, which combines Lasso and ridge regression, selected miRNAs for building the different miRNA biological age biomarkers. The coefficients from the selection were used to calculate miRNA Age (mirAge), miRNA PhenoAge (mirPA), miRNA FI (mirFI), and miRNA Mortality (mirMort) in the test set (*n* = 772) and younger validation set (*n* = 754). The elastic net models were applied to the residuals of the 591 well-expressed log2(CPM) miRNAs resulting from linear regressions on each of them with sex, cell count, RS-subcohort, plate number, and inner/outer well as independent variables. This approach follows the methodology outlined by Huan et al. [23]. We used PCA plots to confirm that differences in miRNA expression levels were no longer driven by technical variation (Additional file 3). In the case of PhenoAge, FI, and all-cause mortality, we additionally included chronological age as an independent variable as we were interested in the adverse health risk beyond chronological age. We optimized the regularization parameters for the elastic net model using tenfold cross-validation and reported the final hyperparameters in Additional file 4. We assessed whether the participant’s biological age as determined by each of the four biomarkers was decelerated or accelerated compared to their chronological age by using the raw residual obtained from regressing chronological age on the miRNA biomarkers of biological age. We used these residuals, the age-accelerated (AA) miRNA-based aging biomarkers, in the downstream analyses. Spearman’s rank correlation was used to assess the correlation between chronological age, the miRNA-based aging biomarkers, and the age-accelerated miRNA-based aging biomarkers.

### Associations of accelerated miRNA biological age with cross-sectional outcomes, mortality, and morbidity

To improve the comparability of estimates describing associations of the four biomarkers with the outcomes of interest, we applied *Z*-transformation to the age-accelerated miRNA-based aging biomarkers. We determined the association of each biomarker with FI, delta frailty, and PhenoAge in the test set and with physical functioning (BADL and IADL) in the test set and younger validation set using linear regression analyses employing the R *stats* package, adjusting for age, sex, cell counts, RS-subcohort, inner/outer well, plate number, and in the case of delta frailty, baseline FI. In the younger validation cohort, we also determined associations between scaled age-accelerated aging biomarkers with self-reported health compared to peers as the reference category, using multinomial logistic regression. These analyses were performed using the R-package *nnet* and included adjustments for age, sex, cell counts, inner/outer well measurements, and plate numbers. We examined associations between each age-accelerated miRNA-based aging biomarker and risk of all-cause mortality in the test set and younger validation set and first and multi-morbidity in the test set using Cox proportional hazard models with age at blood sampling to age at censoring as timescale. This timescale was used to control for left censoring as participants had to live up until blood sampling to get selected in the study population. We employed the R *survival* package to construct the Cox proportional hazard models and adjusted our analyses for the same covariates as the linear regression, only removing age as a covariate because of its inclusion in the timescale. We used FDR correction to control for multiple testing [34].

## Results

We analyzed plasma miRNA levels from 1930 Rotterdam Study participants from the first (RS-I) and second (RS-II) subcohorts with successful miRNA measurements and complete data on chronological age and PhenoAge. These participants were 60:40 split into a training and test set. Additionally, plasma miRNA information was available for 754 participants from the fourth subcohort (RS-IV), which served as the validation set (Fig. 1). The mean age was 71.7 years (standard deviation (SD) = 7.5) in the training set (*n* = 1158) and 71.8 years (SD = 7.7) in the test set (*n* = 772), representing an advanced age population. In the validation set (RS-IV), the participants were younger, with a mean age of 56.6 years (SD = 11.5). The characteristics of the study population are summarized in Table 1. There was no significant difference regarding these characteristics compared to the training and test sets.

**Table 1:** Descriptive characteristics of the study population

	**Train set (*n* = 1158)**	**Test set (*n* = 772)**	***P***_**train test**_	**Validation set (*n* = 754)**	***P***_**train validation**_
Age, years (SD)	71.7 (7.5)	71.8 (7.7)	0.75	56.6 (11.5)	< .001
Women, *n* (%)	652 (56.3)	447 (57.9)	0.52	436 (57.8)	0.68
Ancestry^a^			0.10		< .001
European, *n* (%)	1109 (95.8)	739 (95.7)		618 (82.0)	
African, *n* (%)	4 (0.3)	0 (0.0)		54 (7.2)	
Asian, *n* (%)	9 (0.8)	12 (1.6)		21 (2.8)	
Admixed, *n* (%)	9 (0.8)	3 (0.4)		46 (6.1)	
PhenoAge, years (SD)	66.2 (10.7)	66.0 (10.9)	0.87	Not available	
Frailty index (SD)^a^	0.23 (0.10)	0.24 (0.11)	0.14	Not available	
Died, *n* (%)	737 (63.6)	474 (61.4)	0.34	45 (4.0)	< .001
Perceived health, *n* (%)^a^					
Worse than peers	Not available	Not available		119 (41.5)	
Equal to peers				303 (42.0)	
Better than peers				299 (16.5)	
Delta frailty index (SD)	− 0.01 (0.08)	− 0.01 (0.08)	0.56	Not available	
Baseline morbidity, *n* (%)	345 (29.8)	245 (31.7)		Not available	
Baseline multimorbidity, *n* (%)	67 (5.8)	40 (5.2)		Not available	
BADL (SD)^a^	3.5 (3.9)	3.8 (4.2)	0.15	2.5 (3.4)	< .001
IADL (SD)^a^	2.5 (3.6)	2.8 (4.0)	0.21	1.0 (2.3)	< .001

Values are mean (standard deviation) for continuous variables or number (percentage) for categorical variables

*BADL* basic activities of daily living, *IADL* instrumental activities of daily living, *n* number of participants, *P*_train test_ the *p*-value of the *t*-test (continuous outcomes) or *χ*^2^ test (binary outcomes) between the baseline characteristics of the training and test set, *P*_train validation_ the *p*-value of the *t*-test (continuous outcomes) or *χ*^2^ test (binary outcomes) between the baseline characteristics of the training and validation set, *SD* standard deviation

^a^Indicates missing data: Ancestry (27 participants in training set, 18 test, 15 validation), frailty (13 training, 13 test), BADL (21 training, 18 test, 2 validation), IADL (356 training, 242 test, 166 validation), and self-perceived health (33 participants)

A total of 591 out of 2083 circulatory miRNAs were found to be well-expressed in plasma; the list of these miRNAs is provided in Additional file 5. We updated our previous pipeline, which selected well-expressed miRNAs using the lower limit of quantification method on log2(CPM) and applied linear regression for differential expression analysis. The updated pipeline incorporates plate and inner/outer well position correction and uses DESeq2, which includes size factor normalization, applied to raw counts. With this improved approach, we identified 188 miRNAs showing significant differential expression (FDR-corrected) with chronological age in both the training and test sets (Additional file 6). Similarly, 227 miRNAs were associated with PhenoAge, 61 with the frailty index (FI), and 16 with 10-year mortality independent of chronological age in both the training and test sets, after FDR correction (Additional file 5). Further validating the results for chronological age, we conducted an analysis in the younger cohort (RS-IV), where 177 miRNAs were significantly replicated after FDR correction. All associations were in the same direction across the training, test, and, when applicable, validation set (Additional file 5). Volcano plots for the associations are displayed in Additional file 6. The results were consistent across sensitivity analyses, wherein we used different cut-off values for well-expressed miRNAs (Additional file 7). Six miRNAs, namely miR-1287-5p, miR-197-5p, miR-3141, miR-4534, miR-6887-5p, and miR-7107-5p, were upregulated with all four outcomes. Multi-tissue miRNAs were upregulated with age but downregulated with PhenoAge, the frailty index, and ten-year mortality. Furthermore, kidney-specific miRNAs were overexpressed with PhenoAge, spleen-specific miRNAs with PhenoAge, FI, and 10-year mortality, brain- and bone-specific miRNAs with FI, and tongue-specific miRNAs with mortality. Ardenal gland-specific miRNAs were underrepresented in the miRNAs significantly associated with chronological age, PhenoAge, and FI, and brain-specific miRNAs were underrepresented in miRNAs significantly associated with chronological age and mortality. (Additional file 8). Additionally, we observed an overrepresentation of miRNA target genes synapse-related GO terms across all four outcomes (Additional file 9, Additional file 10: Figs. S3a,b–S6a,b). In KEGG pathway analyses, the strongest overrepresentation of miRNA target genes was seen for cancer pathways with chronological age, endocytosis with PhenoAge, the MAPK signaling pathway with FI, and the RAS signaling pathway with ten-year mortality (Additional file 10: Figs. S3c,d–S6c,d, Additional file 10).

**Fig. 2:**
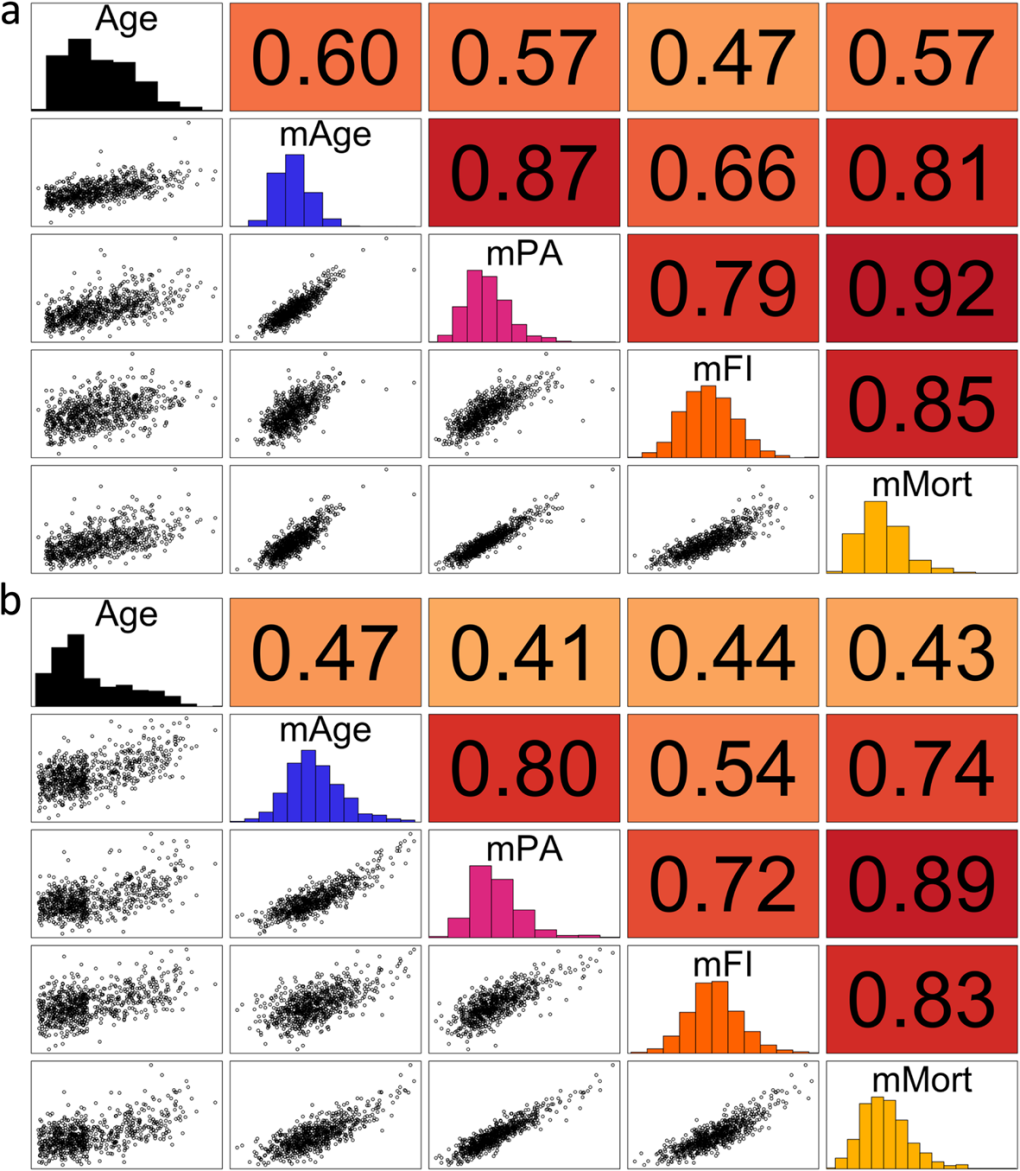
Spearman’s correlations between miRNA Age and chronological age in the test and validation set. MirAge indicates miRNA Age; mirPA, miRNA PhenoAge; mirFI, miRNA frailty index (FI); mirMort, miRNA Mortality. Values represent Spearman’s rank correlation in **a** the test set and **b** the validation set; the background color is darker for higher correlations

Next, we built four miRNA-based aging biomarkers in the training set trained on chronological age, PhenoAge, FI, and mortality using elastic net models. This is a regularized regression technique that combines both Lasso and Ridge methods to improve prediction accuracy and handle correlated variables. This approach allowed us to generate four aging biomarkers that capture distinct aspects of biological aging. The resulting biomarkers—mirAge, mirPA, mirFI, and mirMort—comprised 108, 153, 81, and 50 miRNAs, respectively. Detailed information on the specific miRNAs selected for each biomarker and their corresponding weights is available in Additional file 12. Additional file 13 represents the overlap between the miRNAs selected for the different miRNA-based aging biomarkers. All miRNA-based aging biomarkers showed moderate correlations with chronological age, with Spearman’s rank correlation coefficients (*r*) in the test set ranging from 0.47 for mirFI to 0.60 for mirAge. Concerning the raw biomarkers, mirPA and mirMort showed the highest correlation (*r* = 0.92), while mirAge and mirFI displayed the lowest (*r* = 0.66). Correlation patterns were similar in the test and validation set and among the age-accelerated biomarkers (Fig. 2, Additional file 14).

**Fig. 3:**
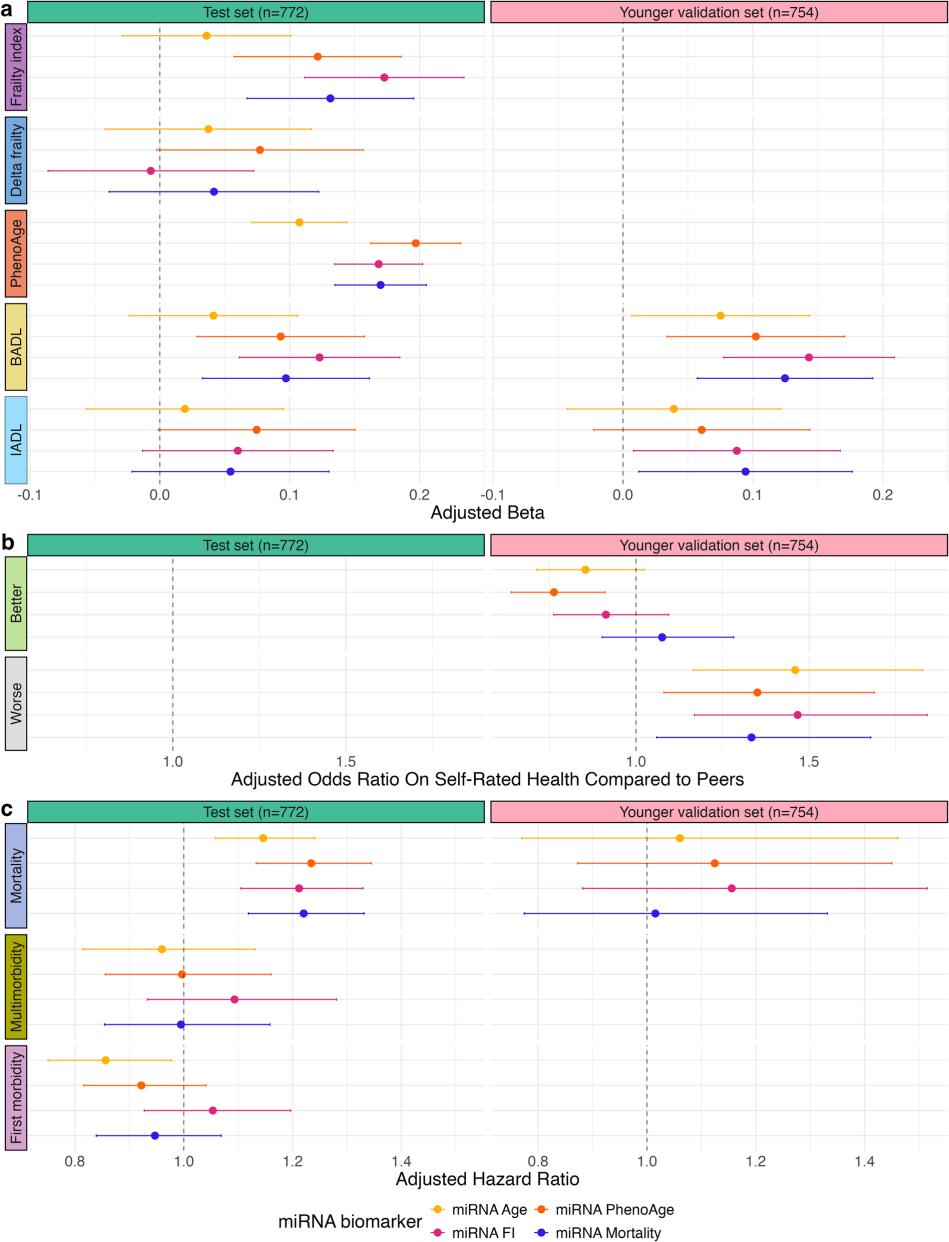
Associations of miRNA-based aging biomarkers with age-related health outcomes in test and validation set. The figure represents a one-standard-deviation increase in miRNA-based aging biomarkers on several health-related outcomes. **a** Association with frailty index, delta frailty, PhenoAge, basic activities of daily living (BADL), and instrumental activities of daily living (IADL) presented as beta coefficients and 95% confidence intervals. **b** Odds of reporting better or worse health than peers, presented as odds ratios and 95% confidence intervals. **c** Risk of mortality, multi-morbidity, and first morbidity, presented as hazard ratios and 95% confidence intervals. The left panels display results in the test set, while the right panels show results in the validation set

Since aging biomarkers are hypothesized to reflect age-related health risks beyond their training outcomes, we assessed the associations of the miRNA-based aging biomarkers with various age-related health outcomes. Figure 3a illustrates the cross-sectional associations of scaled age-accelerated miRNA-based aging biomarkers with frailty, delta frailty, BADL, and IADL in both the test and younger validation sets. Elevated age-accelerated miRNA biomarker scores showed associations with various cross-sectional aging phenotypes. Unless otherwise specified, we report the false discovery rate significant associations. Specifically, age-accelerated mirFI, mirMort, and mirPA were associated with the frailty index (beta-coefficient per SD increase ranged 0.17 (95% confidence interval: 0.11; 0.23) for mirFI, 0.13 (0.07; 0.20) for mirMort, and 0.12 (0.06; 0.19) for mirPA). Additionally, while not statistically significant, higher miRNA-based aging biomarkers were associated with an increase in frailty index scores at the next visit (~ 7 years later), independent of cross-sectional frailty index scores, with the highest observed effect estimates for mirPA (0.08 (0.00; 0.16)). All four biomarkers were associated with higher PhenoAge, with the smallest effect estimate observed for mirAge (0.11 (0.07; 0.14)) and the largest for mirPA (0.20 (0.16; 0.23)). Moreover, mirPA, mirFI, and mirMort showed associations with subjective physical functioning with a focus on locomotive health as measured by BADL in the test set (mirPA: 0.09 (0.03; 0.16), mirFI: 0.12 (0.06; 0.18), mirMort: 0.10 (0.03; 0.16)) as well as in the younger validation set (mirPA: 0.10 (0.03; 0.17), mirFI: 0.14 (0.08; 0.21), mirMort: 0.12 (0.06; 0.19)). Similarly, elevated scores were associated with more problems with subjective physical functioning with a focus on cognitive health as measured by IADL, albeit without statistical significance. The highest effect estimate was observed for mirPA (0.07 (0.00; 0.15)) in the test set for mirMort (0.09 (0.01; 0.18)) in the validation set. All effect estimates can be found in Additional file 15. Furthermore, elevated age-accelerated miRNA-based aging biomarkers were associated with higher odds of self-reported health worse than peers (all four biomarkers ranging per SD increase from 1.33 (1.06; 1.68) for mirAge to 1.47 (1.17; 1.84) for mirPA). Additionally, a standard deviation increase in mirFI was also associated with lower odds of self-reported health better than peers (0.76 (0.64; 0.91)), as shown in Fig. 3b and Additional file 16.

Using the developed miRNA-based aging biomarkers, we tested their ability to predict risks of all-cause mortality in the test and younger validation set, as well as first and multi-morbidity in the test set (Fig. 3c, Additional file 17). Elevated scores on all four scaled age-accelerated miRNA-based aging biomarkers were associated with an increased risk of mortality (median follow-up time of 16.1 years) in the test set, ranging between a hazard ratio per SD increase of 1.15 (1.06;1.24) for mirAge and 1.23 (1.13; 1.34) for mirPA. These findings were robust to adjustment for FI or PhenoAge at time of blood sampling (Additional file 18). In the younger validation set, with a shorter follow-up time (median of 5.4 years), we observed a similar trend with hazard ratios per SD increase ranging between 1.02 (0.77; 1.33) for mirMort to 1.16 (0.88; 1.52) for mirFI. Conversely, no associations were observed between miRNA-based aging biomarkers and higher risk of first morbidity or multi-morbidity (with hazard ratios per SD increase ranging from respectively 0.86 (0.75; 0.98) for mirAge to 1.05 (0.93; 1.20) for mirFI and 0.96 (0.81; 1.13) for mirAge to 1.09 (0.93; 1.28) for mirFI).

## Discussion

In this population-based cohort, we developed four plasma miRNA-based aging biomarkers based on chronological age, PhenoAge, FI, and all-cause mortality. These miRNA-based aging biomarkers were associated with being and becoming frailer, lower subjective physical functioning, worse self-reported health, and all-cause mortality, but not with morbidity. Biomarkers trained on outcomes other than chronological age captured the risk of adverse health outcomes better than miRNA age.

The unique miRNA dataset, characterized by its comprehensive inclusion of more than 2000 plasma circulating miRNAs and substantial sample size in the Rotterdam Study cohort, enabled us to describe a distinct plasma miRNA signature in both an advanced-aged and middle-aged cohort. Our findings, using an updated pipeline to minimize technical biases, validate the results of our previous study using the old pipeline [24]. All top 20 chronological age-related miRNAs identified in the prior analysis retained robust associations in the current study. However, among the top 20 miRNAs associated with PhenoAge, miR-106b-5p, miR-16-5p, miR-92a-3p, and miR-25-3p did not show robust associations in the current study. We urge future studies to use the current results in future meta-analyses.

Of the top 20 miRNAs associated with chronological age in the test set, 11 were also among the top 20 miRNAs in the younger validation set. These include miRNAs previously reported as age-related, namely: miR-17-5p [20, 40], miR-19a-3p [20, 23, 41–43], miR-19b-3p [43, 44], miR-93-5p [40, 45], and miR-146a-5p [20, 23, 40], miR-185-5p [20, 23, 45], miR-197-5p [20], and miR-425-5p [20]. Additionally, miR-345-5p has previously been identified in the Framingham Heart Study as associated with chronological age [20, 23]; however, this finding was not replicated in a smaller study [40]. Lastly, we observed miR-6887-5p as a novel miRNA in association with chronological age. Direct comparisons of effect sizes and directions across studies are hindered by the substantial heterogeneity in sequencing techniques and target panels across studies. To advance the field, harmonization of miRNA profiling methods and target panel selection is urgently needed to enable meaningful comparisons across studies.

Furthermore, we were the first to determine the association of plasma miRNAs with frailty and ten-year mortality in a population-based setting. We observed upregulation of miR-1287-5p, miR-197-5p, miR-3141, miR-6887-5p, and miR-7107-5p with all four outcomes. Just as miR-197-5p [20], miR-1287-5p has been previously associated to chronological age [40], while miR-7107-5p showed moderate correlation with age in prior research [19]. MiR-3141 has been associated with downregulation in age-related white matter lesions [46], and miR-6887-5p has been linked to prostate cancer [47].

Our analysis of tissue specificity of the identified miRNAs revealed overexpression of multi-tissue miRNAs associated with chronological age and under expression of those linked to PhenoAge, frailty, and ten-year mortality. Yet, we observed distinct organ-specific miRNA expression patterns such as overrepresentation of kidney-specific miRNAs with PhenoAge and spleen-specific miRNAs with frailty and mortality. These findings suggest a dual role for miRNAs in systemic aging processes and organ-specific pathways linked to age-related health outcomes. As expected, given the broad nature of aging, the overrepresentation of miRNA target genes in synapse-related GO terms across outcomes emphasizes the involvement of neural pathways, while KEGG pathway analyses point to cancer, endocytosis, and MAPK and RAS signaling as key involved mechanisms.

In this study, we trained four plasma miRNA-based aging biomarkers. These miRNA-based aging biomarkers were trained not solely on chronological age but—as we were interested in biomarkers predicting unhealthy aging—also on the frailty index, PhenoAge, and mortality. Higher scores on these miRNA-based aging biomarkers were associated with higher mortality risk, frailty, increase in frailty, and lower self-reported health and physical functioning. However, these biomarkers did not reflect increased risk on all-cause first and multi-morbidity. This could reflect the complex and multifaceted nature of aging, where biomarkers may not directly capture morbidity. Alternatively, the lack of association may be due to the exclusion of participants with prevalent disease, which might have limited the diversity of the sample and reduced the sensitivity of the biomarkers to detect associations with morbidity outcomes. Notably, mirPA, mirFI, and mirMort outperformed mirAge in the association with mortality, frailty, self-reported health, and subjective physical functioning. This finding is in line with earlier reports on epigenetic and metabolomics-based biomarkers of biological age [8, 9, 11], thereby contributing to the growing body of evidence that aging biomarkers trained on health-related information outperform aging biomarkers trained on chronological age. Although mirPA, mirFI, and mirMort are highly correlated and share several miRNAs, they represent different constructs: mirPA as a miRNA-proxy for PhenoAge, mirFI for the frailty index, and mirMort for mortality risk. All three biomarkers are associated with all-cause mortality independent of baseline PhenoAge and frailty index. This indicates that while PhenoAge and the frailty index are linked to all-cause mortality, the miRNA-based aging biomarkers provide additional predictive value beyond these metrics. Thus, we propose that the associations observed for the miRNA-based aging biomarkers reflect underlying biological aging, rather than solely the outcome on which they were trained. The lower performance of mirFI in association with delta FI as compared to other biomarker-outcome associations of mirFI might be a result of the adjustment for FI at blood collection as mirFI was trained on FI at blood collection.

While the miRNA-based aging biomarkers were associated with lower self-reported health and physical functioning in the younger validation cohort, suggesting they reflect early biological aging processes linked to declining health, they did not show consistent associations with mortality. These discrepancies may be due to several factors, including limited statistical power for the mortality analyses in the younger cohort (*n* = 45) with a shorter follow-up period (median of 5.4 years in RS-IV compared to 16.1 years in RS-I and RS-II). Additionally, variations in biomarker effects across different age groups could contribute to these inconsistencies. Therefore, future studies with larger, more diverse cohorts and longer follow-ups are needed to clarify these findings. Additionally, external validation in independent, larger cohorts is also necessary to better understand the relationship between miRNA-based aging biomarkers, morbidity, and mortality.

Our study has some limitations worth mentioning. Firstly, we were unable to identify an independent large-scale population-based cohort with similar cell-free miRNA data to validate our results. Therefore, we have conducted cross-validation across three different RS subcohorts and established internal validity. Importantly, we validated our score in two independent sets: a test set with the same age range as the discovery cohort and a validation set with a different age range. While merging all participants and randomly splitting the total sample into discovery, test, and validation sets with similar age distributions would have been ideal, this was not feasible due to RS-IV subcohort being shipped in a separate batch from the RS-I and RS-II subcohorts. Nevertheless, we demonstrated the associations beyond the original batch and age differences, thereby strengthening our validation. Secondly, we deemed our study population too small to perform sex-specific analyses. Therefore, we adjusted all analyses for sex and urged future studies to explore whether sex-specific miRNA-based aging biomarkers outperform the non-sex-specific biomarkers from the current study. Thirdly, the RS cohorts predominantly consist of individuals of European ancestry. As a result, the generalizability of these biomarkers to other ancestral groups remains uncertain. Future studies involving more diverse populations are essential to evaluate the applicability and validity of these biomarkers across different ethnic and genetic backgrounds. Furthermore, we did not have the same outcome data available across cohorts; RS-IV lacked data for two out of the four primary outcomes: PhenoAge and the frailty index. However, as aging biomarkers aim to capture a holistic measure of aging and should therefore provide information beyond their primary outcome aging, we believe we were able to evaluate the performance of the four miRNA-based aging biomarkers in the validation set by their associations to indicators of unhealthy aging that we used as secondary outcome measures. Of the secondary outcomes, the follow-up for the morbidity outcomes was limited to two subcohorts, RS-I and RS-II, and unavailable in RS-IV. Information on self-reported health was available in RS-IV but not in RS-I and RS-II. Yet, we observed consistent effect estimates of the associations with BADL and IADL across the test and validation sets, indicating potential generalizability of the results to younger age groups. Furthermore, although the dataset includes information on over 2000 miRNAs, it is a targeted dataset, which limits the ability to identify potentially relevant miRNAs that were not preselected as targets. Finally, we were unable to assess the correlation of the miRNA-based aging biomarkers to biological aging biomarkers based on other omics data, such as proteomics, metabolomics, messenger-RNAs, and DNA methylation, because data were not available at the same examination. Nor were we able to systematically compare the advantages and disadvantages of the different aging biomarkers. We recommend that future research should incorporate multi-omics approaches to explore the interactions between these various aging biomarkers, thereby advancing our understanding of the complex nature of biological aging. A major strength of our study is the usage of a large community-based follow-up study data with a novel RNA-sequencing method to assay the levels of a wide array of cell-free miRNAs in stored plasma samples. The mortality and morbidity outcomes were well-ascertained. Furthermore, miRNA-based aging biomarkers were able to identify health-related outcomes in an overall healthy population, emphasizing their potential for early detection of individuals at risk for age-related health issues. Lastly, we had information on a large variety of outcome measures providing a better insight into the complexity of the aging process.

## Conclusions

Collectively, the current study indicates plasma-based cell-free miRNA signatures of aging phenotypes and miRNA-based aging biomarkers in both advanced-aged and middle-aged population cohorts. Furthermore, this study shows that miRNA biomarkers for biological age can reflect (un)healthy aging, as they are associated with age-related decline and mortality irrespective of chronological age. Further research is needed to compare miRNA-based aging biomarkers with those from other molecular layers and improve the multi-biomarker approach for monitoring aging-related diseases.

## Supplementary Information

Additional file 1: Table S1. Overview of the adapted frailty index, included deficits, and used cut-off values.

Additional file 2: Table S2. Ascertainment of major morbidities in the Rotterdam Study.

Additional file 3: Figure S1. PCA of miRNA expression levels adjusted for technical variation.

Additional file 4: Table S3. Hyperparameters and performance in training and test sets.

Additional file 5. Differential expression with chronological age, PhenoAge, the frailty index, and ten-year mortality for 591 well-expressed microRNAs across training, test, and validation sets.

Additional file 6: Figure S2. Differential expression of chronological age, PhenoAge, frailty index, and ten-year mortality.

Additional file 7. Differential expression with chronological age using different cut-offs for well-expressed microRNAs in the training set.

Additional file 8. Overrepresentation analyses with different tissues of microRNAs differentially expressed with chronological age, PhenoAge, the frailty index, and ten-year mortality.

Additional file 9. Overrepresentation analyses of GO terms for microRNAs differentially expressed with chronological age, PhenoAge, the frailty index, and ten-year mortality.

Additional file 10: Figures S3–S6. Biological pathways overrepresented among differentially expressed miRNAs. Results include overrepresentation analyses for biological process GO terms (a, b) and KEGG pathways (c, d) based on target genes of miRNAs associated with ten-year mortality. Panels a and c display dot plots with all results, while panels b and d show the corresponding Gene-Concept networks.

Additional file 11. Overrepresentation analyses of KEGG terms for microRNAs differentially expressed with chronological age, PhenoAge, the frailty index, and ten-year mortality.

Additional file 12. Coefficients of miRNA-based biomarkers (mirAge, mirPA, mirFI, and mirMort).

Additional file 13: Figure S7. Overlap between miRNAs selected in miRNA-based aging biomarkers.

Additional file 14: Figure S8. Spearman’s correlations between miRNA Age and chronological age in the test and validation sets.

Additional file 15: Table S10. Associations between standardized age-accelerated miRNA aging biomarkers and continuous measure.

Additional file 16: Table S11. Associations of standardized miRNA-based aging biomarkers with self-reported health in the validation set.

Additional file 17: Table S12. Associations between standardized miRNA-based aging biomarkers and first morbidity, multi-morbidity, and mortality.

Additional file 18: Table S13. Associations between standardized miRNA-based aging biomarkers and all-cause mortality with and without adjustment for PhenoAge and the frailty index.

## Data Availability

The code used in this study has been made publicly available on GitHub [48] (https://github.com/liekekuiper/plasma-mirnaaging). MiRNA expression profiles, including the log2CPM-transformed and rounded miRNA counts, along with the participant's chronological age and their assignment to the training, testing, or validation set, has been deposited at the European Genome-phenome Archive (EGA), under the accession number EGAS00001008117, which is hosted by the EBI and the CRG [49]. Access to the data requires submission of an application and a data use agreement to ensure compliance with ethical standards and the protection of participant confidentiality. Further information about EGA and the detailed guidelines for requesting access can be found on https://ega-archive.org (the EGA accession number EGAS00001008117). Additional clinical and covariate data of the participants used in this study can be obtained upon request. Requests should be directed towards the corresponding author (m.ghanbari@erasmusmc.nl) or the management team of the Rotterdam Study (datamanagement.ergo@erasmusmc.nl), which has a protocol for approving data requests. Upon receiving a request, the management team evaluates it typically within one month, and if approved, the requested data will be shared in compliance with the GDPR regulations, after signing a Data Transfer Agreement (DTA).
