# Supplementary material for: Plasma microRNA signatures of aging and their links to health outcomes and mortality: findings from a population-based cohort study
Source: Genome Med. 2025 Jun 25;17:70. doi: 10.1186/s13073-025-01437-5 (PMC12188677; doi:10.1186/s13073-025-01437-5)
Supplement: Supplementary file 1 — Additional file 1: Table S1. Overview of the adapted frailty index, included deficits, and used cut-off values. [file 13073_2025_1437_MOESM1_ESM.docx]

Additional file 1: Table S1. Overview of the adapted frailty index included deficits and used cut-off values.

| # | Item | Item(s) – additional information | Cut-off value | Source & reference |
| --- | --- | --- | --- | --- |
| 1 | Dressing and grooming | Able to get clothes from closets or drawers; able to dress; able to shampoo your hair; able to comb your hair or do your make-up | Without any difficulty = 0  With some difficulty = 0.33  With much difficulty = 0.66  Unable to do = 1 | Stanford Health Assessment Questionnaire [33] |
| 2 | Arising | Able to stand up from a straight chair without using your arms for support; able to get in and out of bed | Without any difficulty = 0  With some difficulty = 0.33  With much difficulty = 0.66  Unable to do = 1 | Stanford Health Assessment Questionnaire [33] |
| 3 | Eating | Able to cut meat and lift a full cup or glass to your mouth; able to open a new carton of milk | Without any difficulty = 0  With some difficulty = 0.33  With much difficulty = 0.66  Unable to do = 1 | Stanford Health Assessment Questionnaire [33] |
| 4 | Walking | Able to walk outdoors on flat ground; able to climb up five steps | Without any difficulty = 0  With some difficulty = 0.33  With much difficulty = 0.66  Unable to do = 1 | Stanford Health Assessment Questionnaire [33] |
| 5 | Hygiene | Able to wash and dry your entire body; able to take a shower/bath | Without any difficulty = 0  With some difficulty = 0.33  With much difficulty = 0.66  Unable to do = 1 | Stanford Health Assessment Questionnaire [33] |
| 6 | Reach | Able to reach and get down a 1kg object from just above your head; able to bend down to pick up clothing from the floor | Without any difficulty = 0  With some difficulty = 0.33  With much difficulty = 0.66  Unable to do = 1 | Stanford Health Assessment Questionnaire [33] |
| 7 | Grip | Able to open a car door? Able to open jars which have been previously opened | Without any difficulty = 0  With some difficulty = 0.33  With much difficulty = 0.66  Unable to do = 1 | Stanford Health Assessment Questionnaire [33] |
| 8 | Riding a bike | Able to ride a bike | Without any difficulty = 0  With some difficulty = 0.33  With much difficulty = 0.66  Unable to do = 1 | Lawton Instrumental Activities of Daily Living scale [34] |
| 9 | Telephone | Able to use the telephone | Without any difficulty = 0  With some difficulty or using a customized phone = 0.33  With much difficulty = 0.66  Unable to do = 1 | Lawton Instrumental Activities of Daily Living scale [34] |
| 10 | Meal | Able to prepare meals | Without any difficulty = 0  With some difficulty = 0.33  With much difficulty = 0.66  Unable to do = 1 | Lawton Instrumental Activities of Daily Living scale [34] |
| 11 | Laundry | Able to do the laundry | Without any difficulty = 0  With some difficulty = 0.33  With much difficulty = 0.66  Unable to do = 1 | Lawton Instrumental Activities of Daily Living scale [34] |
| 12 | Financial | Able to do finances | Without any difficulty = 0  With some difficulty = 0.33  With much difficulty = 0.66  Unable to do = 1 | Lawton Instrumental Activities of Daily Living scale [34] |
| 13 | Depressed affect | I felt that I could not shake off the blues even with help from family or friends; I felt depressed; I thought my life had been a failure; I felt lonely; I had crying spells; I felt sad | Rarely or none of the time = 0  Some or a little of the time = 0.33  Occasionally or a moderate amount of time = 0.66  Most or all of the time = 1 | The CES-D scale: a self-report depression scale [54] |
| 14 | Positive affect | I felt that I was just as good as other people; I felt hopeful about the future; I was happy; I enjoyed life | Rarely or none of the time = 1  Some or a little of the time = 0.66  Occasionally or a moderate amount of time = 0.33  Most or all of the time = 0 | The CES-D scale: a self-report depression scale [54] |
| 15 | Somatic and retarded activity | I did not feel like eating my appetite was poor; I had trouble keeping my mind on what I was doing; I felt that everything I did was an effort; I felt fearful; my sleep was restless; I talked less than usual; I could not get “going” | Rarely or none of the time = 0  Some or a little of the time = 0.33  Occasionally or a moderate amount of time = 0.66  Most or all of the time = 1 | The CES-D scale: a self-report depression scale [54] |
| 16 | Interpersonal | I was bothered by things that usually don’t bother me; people were unfriendly; I felt that people dislike me | Rarely or none of the time = 0  Some or a little of the time = 0.33  Occasionally or a moderate amount of time = 0.66  Most or all of the time = 1 | The CES-D scale: a self-report depression scale [54] |
| 17 | Falling | How often did you fall in the past 12 months? | No falling = 0  Less than once a month = 0.5  More than once a month = 1 |  |
| 18 | Joint complaints | Did you have joint pain or other complaints from the knees, hips, back, or hand? | No = 0  Yes = 1 |  |
| 19 | Mobility | Do you use any support to walk? | No = 0  Walking aid = 0.5  Wheelchair = 1 |  |
| 20 | Forgetfulness | Do you sometimes forget what you were about to do? | No = 0  Yes = 1 |  |
| 21 | Aphasia | Do you have difficulties with finding the right words? | No = 0  Yes = 1 |  |
| 22 | Liver enzymes | ALAS, ALAT, Gamma-glutamyl transpeptidase | All values within the range = 0  One or more abnormal values = 1 | Serum blood measurement; cut-off values derived from the Laboratory guide Erasmus MC |
| 23 | Creatinine |  | Male 65-115 umol/L = 0  Other values = 1  Female 55-90 umol/L = 0  Other values = 1 | Serum blood measurement; cut-off values derived from the Laboratory guide Erasmus MC |
| 24 | Hyperlipidemia | High cholesterol or medication against high cholesterol | Statin use and/or cholesterol >6.5 mmol/L  No statin use and cholesterol 2.9-6.5 mmol/L | Serum blood measurement; cut-off values derived from the Laboratory guide Erasmus MC |
| 25 | HDL |  | HDL ≥ 1.55 = 0  HDL < 1.55 = 1 | Serum blood measurement; cut-off values derived from the Laboratory guide Erasmus MC |
| 26 | Systolic blood pressure | Measure three times, average is taken | Systolic blood pressure 90-140 = 0  Systolic blood pressure 140-160 = 0.5  Systolic blood pressure < 90 = 0.5  Systolic blood pressure > 160 = 1 | Serum blood measurement; cut-off values derived from the Laboratory guide Erasmus MC |
| 27 | MMSE | Mini Mental State Examination | Unimpaired >25 = 0  Impaired ≤ 25 =1 |  |
| 28 | LDST | Letter-Digit Substitution Test: the number of correct digits | Above mean or less than 1SD below mean = 0  One SD below mean = 0.5  Two SD below mean = 1 |  |
| 29 | STROOP | Stroop test | Above mean or less than 1SD above mean = 0  One SD above mean = 0.5  Two SD above mean = 1 |  |
| 30 | WFT | Word Fluency test | Above mean or less than 1SD below mean = 0  One SD below mean = 0.5  Two SD below mean = 1 |  |
| 31 | Cancer |  | No = 0  Yes = 1 |  |
| 32 | Lung condition (COPD/Asthma) |  | No = 0  Yes = 1 |  |
| 33 | Cardiovascular diseases |  | No = 0  Yes = 1 | Prevalent CHD [55] |
| 34 | Stroke |  | No = 0  Yes = 1 | Prevalent stroke [55] |
| 35 | Diabetes Mellitus |  | No = 0  High glucose= 0.5  Yes = 1 | Prevalent DM and/or glucose levels |
| 36 | BMI | Body mass index  BMI < 18.5 = underweight  BMI ≤25 and ≥18.5 = normal weight  BMI <25 and ≤30 = overweight  BMI <30 = obese | Normal weight= 0  Overweight = 0.5  Obese or underweight = 1 |  |
| 37 | Hospital admission | Last 12 months | No = 0  Yes = 1 |  |
| 38 | Age-related macular degeneration | Fundus photography after pharmacologic mydriasis. The eyes of each participant were graded and classified separately, and the eye with the more severe grade was used to classify the person. | 0 = 5-year risk of developing advanced AMD in at least one eye is 0.5%  0.25 = 5-year risk is 3% 0.50 = 5-year risk is 12%  0.75 = 5-year risk is 25%  1= 5-year risk is 50% | [56] |
