# Supplementary material for: Plasma microRNA signatures of aging and their links to health outcomes and mortality: findings from a population-based cohort study
Source: Genome Med. 2025 Jun 25;17:70. doi: 10.1186/s13073-025-01437-5 (PMC12188677; doi:10.1186/s13073-025-01437-5)
Supplement: Supplementary file 2 — Additional file 2: Table S2. Ascertainment of major morbidities in the Rotterdam Study. [file 13073_2025_1437_MOESM2_ESM.docx]

Additional file 2: Table S2. Ascertainment of major morbidities in the Rotterdam Study

| Dementia  Participants were screened for dementia at baseline and subsequent center visits. Those with a Mini-  Mental State Examination score < 26 or Geriatric Mental Schedule score > 0 underwent further  investigation and informant interview, including the Cambridge Examination for Mental Disorders of  the Elderly. In addition, the entire cohort was under continuous surveillance for dementia through  electronic linkage of the study database with medical records from general practitioners and the  regional institute for outpatient mental health care. A consensus panel led by a consultant neurologist  established the final diagnosis according to standard criteria for dementia (DSM-III-R),  Alzheimer’s disease (NINCDS–ADRDA) and vascular dementia (NINDS-AIREN). |
| --- |
| Coronary heart disease  Prevalent coronary heart disease (CHD) was defined when the participant suffered a myocardial infarction or underwent a coronary artery bypass grafting or percutaneous coronary revascularization procedure. |
| Cancer  The occurrence of any solid cancer was determined through information obtained by 4-yearly follow-up rounds from the general practitioners (including discharge letters from hospitals) and by linkage with a nationwide registry of histo- and cytopathology in The Netherlands, Pathologisch-Anatomisch Landelijk Geautomatiseerd Archief (PALGA). Two research physicians independently assessed the first date and diagnosis of cancer. All events are pathology-based and were classified according to the International Classification of Diseases (ICD) 10^th^ edition. In case of discrepancy, consensus was sought, or a cancer epidemiologist decided. |
| Diabetes  Prevalent diabetes mellitus type 2 was identified according to the World Health Organization criteria: fasting glucose levels of ≥7.0 mmol/L, non-fasting glucose levels ≥11.1 mmol/L, or the use of glucose-lowering medication. Information regarding the use of glucose-lowering medication was obtained from pharmacy records and home interviews. |
| Stroke  Stroke was defined according to the World Health Organization definition as a syndrome of rapidly developing clinical signs of focal or global disturbance of cerebral function, with symptoms lasting 24 hours or longer or leading to death, with no apparent cause other than of vascular origin. We assessed the prevalence of stroke at baseline during an interview and verified it using medical records. |
| Chronic Obstructive Pulmonary Disease  The diagnosis and classification of COPD was based on the Global Initiative for Chronic Obstructive Lung Disease (GOLD) criteria (proportion of the forced vital capacity (FVC) exhaled in the first second (forced expiratory volume in 1 s (FEV1)/FVC ratio) <70%). |
