# Supplementary material for: Plasma microRNA signatures of aging and their links to health outcomes and mortality: findings from a population-based cohort study
Source: Genome Med. 2025 Jun 25;17:70. doi: 10.1186/s13073-025-01437-5 (PMC12188677; doi:10.1186/s13073-025-01437-5)
Supplement: Supplementary file 3 — Additional file 3: Figure S1. PCA of miRNA expression levels adjusted for technical variation. [file 13073_2025_1437_MOESM3_ESM.docx]

Additional file 3 Figure S1. PCA of miRNA Expression Levels Adjusted for Technical Variation.

**
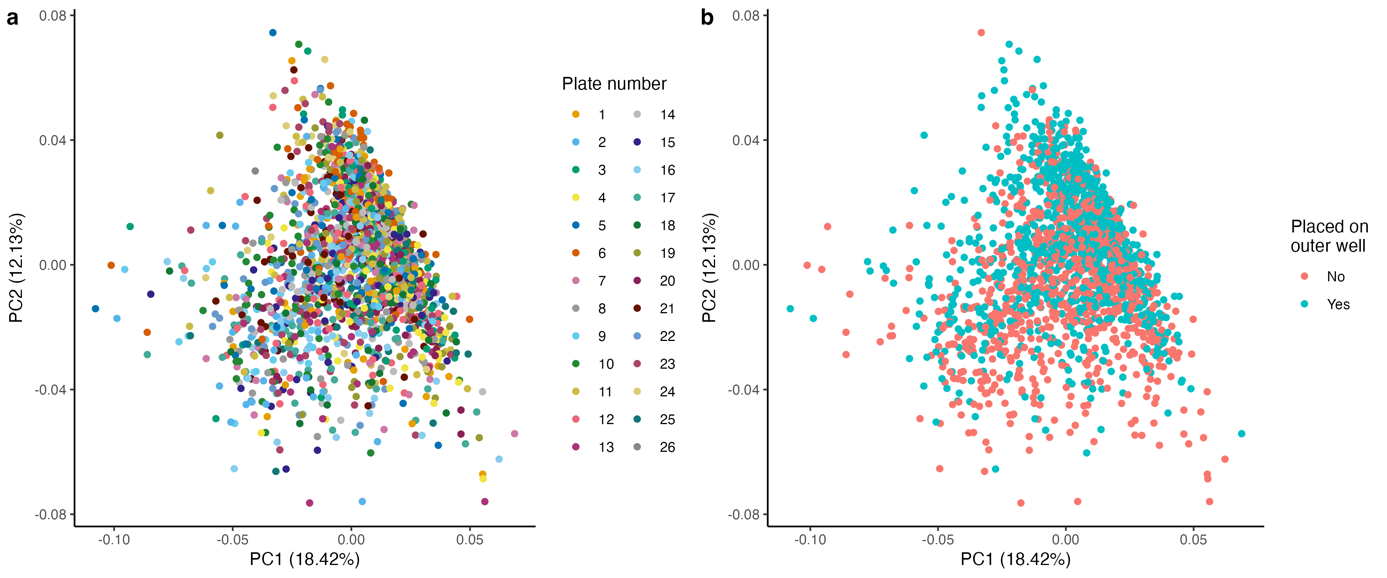
**

The figures display the first (x-axis) and second (y-axis) principal components from a principal component analysis of miRNA expression levels for the 591 well-expressed miRNAs, adjusted for sex, Rotterdam Study sub-cohort, monocyte, lymphocyte counts and red blood cell count, inner/outer well position, and sequencing plate number. Dots in **a.** are colored according to the sequencing plate number, and in **b.** according to whether the sample is positioned in the inner or outer well.
