# Supplementary material for: Plasma microRNA signatures of aging and their links to health outcomes and mortality: findings from a population-based cohort study
Source: Genome Med. 2025 Jun 25;17:70. doi: 10.1186/s13073-025-01437-5 (PMC12188677; doi:10.1186/s13073-025-01437-5)
Supplement: Supplementary file 4 — Additional file 4: Table S3. Hyperparameters and performance in training and test sets. [file 13073_2025_1437_MOESM4_ESM.docx]

Additional file 4: Table S3. Hyperparameters and performance in training and test set

|  | Hyperparameters | | Train set | | | Test set | | |
| --- | --- | --- | --- | --- | --- | --- | --- | --- |
|  | Alpha | Lambda | MSE | RMSE | R^2^ | MSE | RMSE | R^2^ |
| Age | 0.8 | 0.18 | 30.0 | 5.5 | 0.48 | 37.5 | 6.1 | 0.36 |
| PhenoAge | 0.5 | 0.32 | 49.9 | 7.1 | 0.57 | 65.4 | 8.1 | 0.45 |
| Frailty index | 0.3 | 0.01 | 0.01 | 0.09 | 0.24 | 0.01 | 0.10 | 0.15 |
|  |  |  | C | -2 Log Partial Likelihood Deviance | | C | -2 Log Partial Likelihood Deviance | |
| Mortality | 0.3 | 0.13 | 0.70 | 9083.4 | | 0.70 | 5794.5 | |

C indicates concordance; MSE, minimal square error, RMSE, root minimal square error, R^2^ coefficient of determination
