# Supplementary material for: Plasma microRNA signatures of aging and their links to health outcomes and mortality: findings from a population-based cohort study
Source: Genome Med. 2025 Jun 25;17:70. doi: 10.1186/s13073-025-01437-5 (PMC12188677; doi:10.1186/s13073-025-01437-5)
Supplement: Supplementary file 6 — Additional file 6: Figure S2. Differential expression of chronological age, PhenoAge, frailty index, and ten-year mortality. [file 13073_2025_1437_MOESM6_ESM.docx]

Additional file 6 Figure S2. Differential expression of chronological age, PhenoAge, frailty index and 10-year mortality


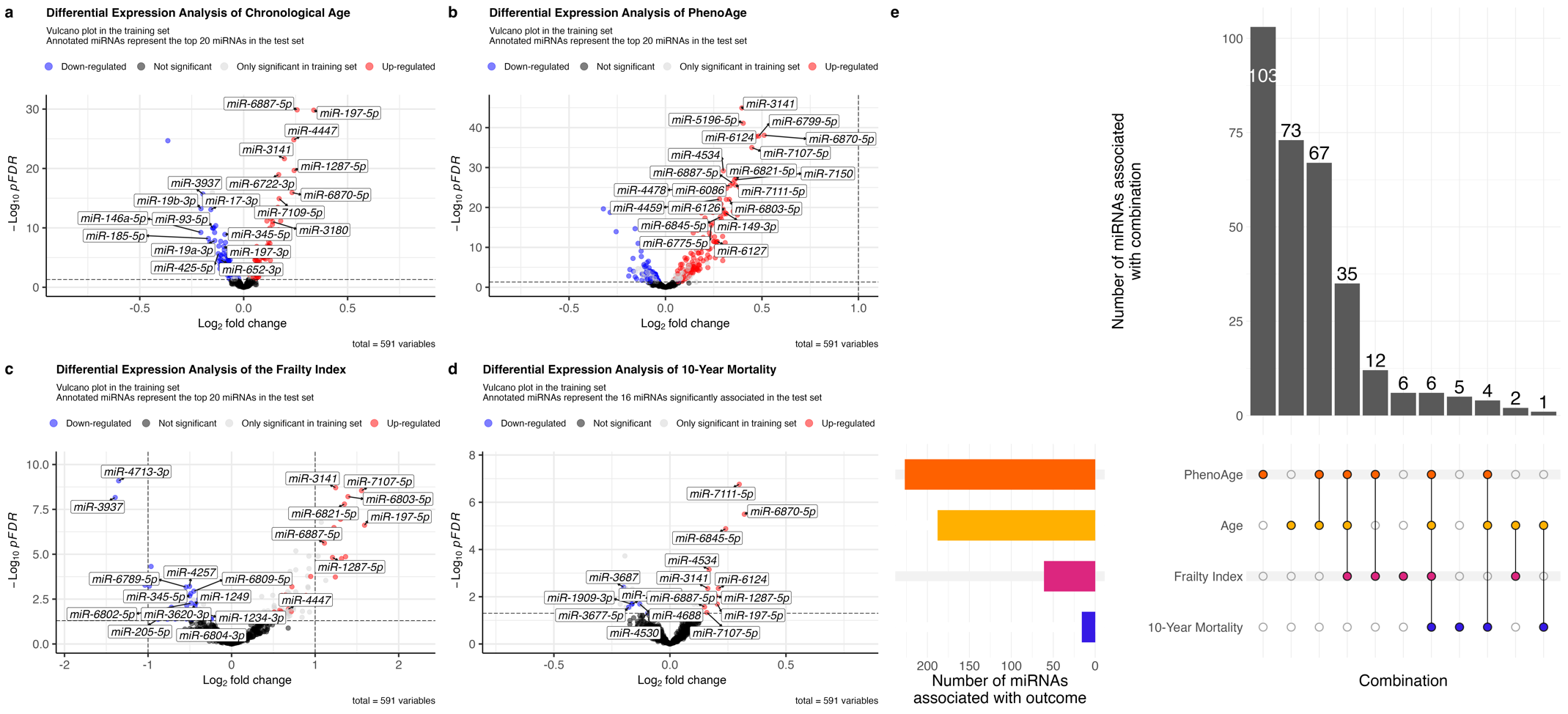
The figure illustrates the expression of 591 well-expressed miRNAs with **a)** chronological age, **b)** PhenoAge, **c),** the frailty index, and **d)** 10-year mortality with adjustment for sex, cell counts, technical covariates, RS-subcohort, and, in the case of the latter three, chronological age in 1,158 participants from the training set of the Rotterdam Study. The value on the x-axis indicates the Log2 fold change and the y-axis the -log10 false discovery rate (FDR) corrected p-value. Dark grey: insignificant (false discovery rate corrected p-value>0.05 in training set); light grey: differentially expressed in the training set, not in the test set (false discovery rate corrected p-value >0.05 in the test set) Blue: differentially expressed (false discovery rate corrected p-value <0.05 in both test and training set; effect size negative); Red: differentially expressed (false discovery rate corrected p-value <0.05 in both test and training set; effect size positive). In **e)** the overlap between differentially expressed miRNAs between the different outcomes is shown. The colored bar plots at the left side demonstrate the number of miRNAs associated with each outcome. The bar plots on top show the number of miRNAs associated with a combination of outcomes, the combinations are shown below when a dot is filled the outcome is included in the combination.
