# Supplementary material for: Plasma microRNA signatures of aging and their links to health outcomes and mortality: findings from a population-based cohort study
Source: Genome Med. 2025 Jun 25;17:70. doi: 10.1186/s13073-025-01437-5 (PMC12188677; doi:10.1186/s13073-025-01437-5)
Supplement: Supplementary file 10 — Additional file 10: Figures S3–S6. Biological pathways overrepresented among differentially expressed miRNAs. Results include overrepresentation analyses for biological process GO terms (a, b) and KEGG pathways (c, d) based on target genes of miRNAs associated with ten-year mortality. Panels a and c display dot plots with all results, while panels b and d show the corresponding Gene-Concept networks. [file 13073_2025_1437_MOESM10_ESM.docx]

Additional file 10 Figures S3-6 Biological pathways overrepresented among differentially expressed miRNAs

**Figure S3.** Biological pathways overrepresented among differentially expressed miRNAs with chronological age


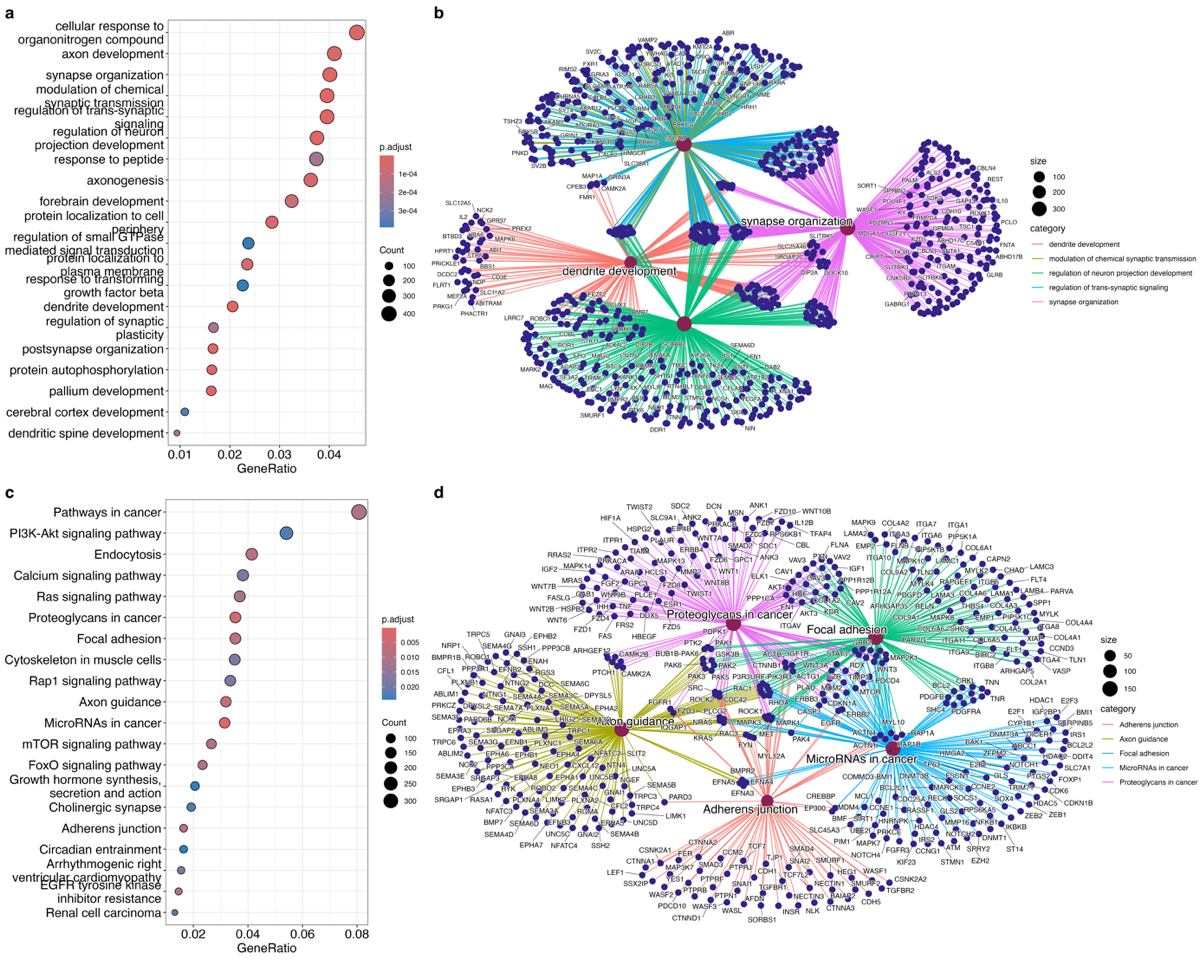


The figure presents the results of overrepresentation analyses for biological process GO terms (**a, b**) and KEGG pathways (**c, d**) based on the target genes of miRNAs differentially expressed with chronological age that had matching gene targets. Panels **a** and **c** display dot plots of the top 20 results, while panels **b** and **d** show the corresponding Gene-Concept networks.

**Figure S4.** Biological pathways overrepresented among differentially expressed miRNAs with PhenoAge


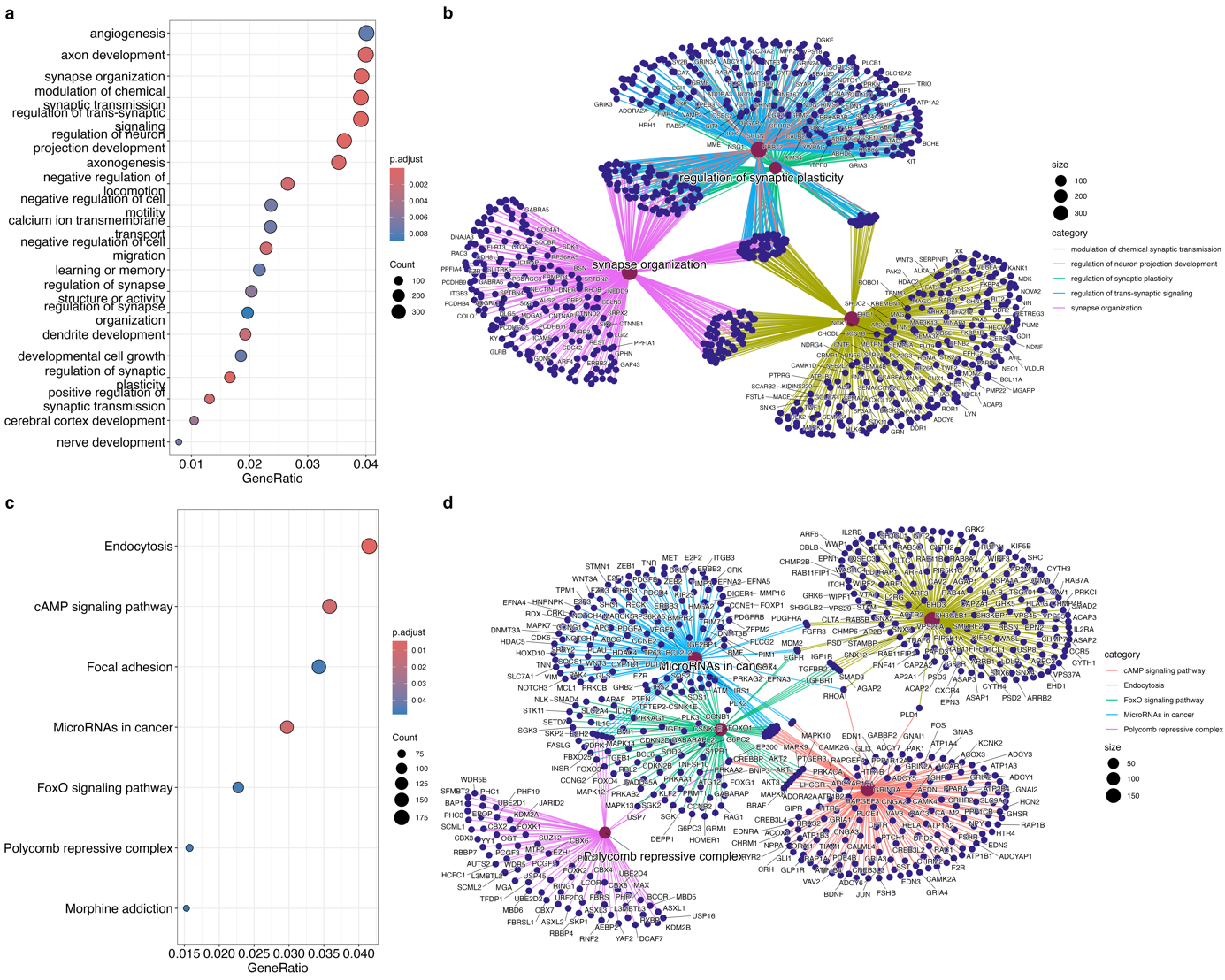


The figure presents the results of overrepresentation analyses for biological process GO terms (**a, b**) and KEGG pathways (**c, d**) based on the target genes of miRNAs differentially expressed with PhenoAge that had matching gene targets. Panel **a** and **c** display dot plots of, respectively the top 20 and all results, while panels **b** and **d** show the corresponding Gene-Concept networks.

**Figure S5.** Biological pathways overrepresented among differentially expressed miRNAs with the frailty index





The figure presents the results of overrepresentation analyses for biological process GO terms (**a, b**) and KEGG pathways (**c, d**) based on the target genes of miRNAs associated with the frailty index. Panels **a** and **c** display dot plots of the top 20 results, while panels **b** and **d** show the corresponding Gene-Concept networks.

**Figure S6.** Biological pathways overrepresented among differentially expressed miRNAs with ten-year mortality


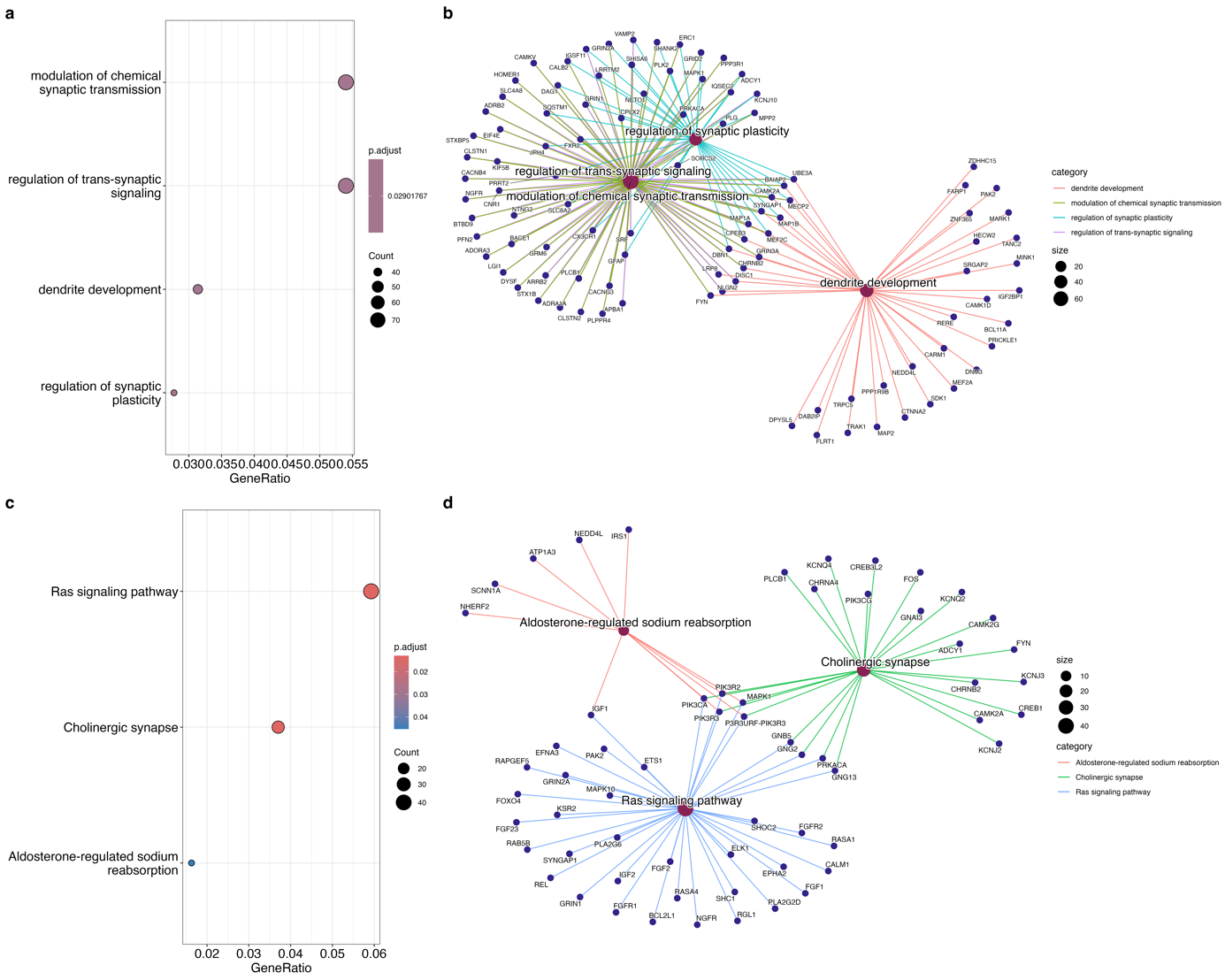


The figure presents the results of overrepresentation analyses for biological process GO terms (**a, b**) and KEGG pathways (**c, d**) based on the target genes of miRNAs differentially expressed with ten-year mortality that had matching gene targets. Panels **a** and **c** display dot plots with all results, while panels **b** and **d** show the corresponding Gene-Concept networks.
