# Supplementary material for: Plasma microRNA signatures of aging and their links to health outcomes and mortality: findings from a population-based cohort study
Source: Genome Med. 2025 Jun 25;17:70. doi: 10.1186/s13073-025-01437-5 (PMC12188677; doi:10.1186/s13073-025-01437-5)
Supplement: Supplementary file 13 — Additional file 13: Figure S7. Overlap between miRNAs selected in miRNA-based aging biomarkers. [file 13073_2025_1437_MOESM13_ESM.docx]

Additional file 13: Figure S7. Overlap between miRNAs selected in miRNA-based aging biomarkers

**
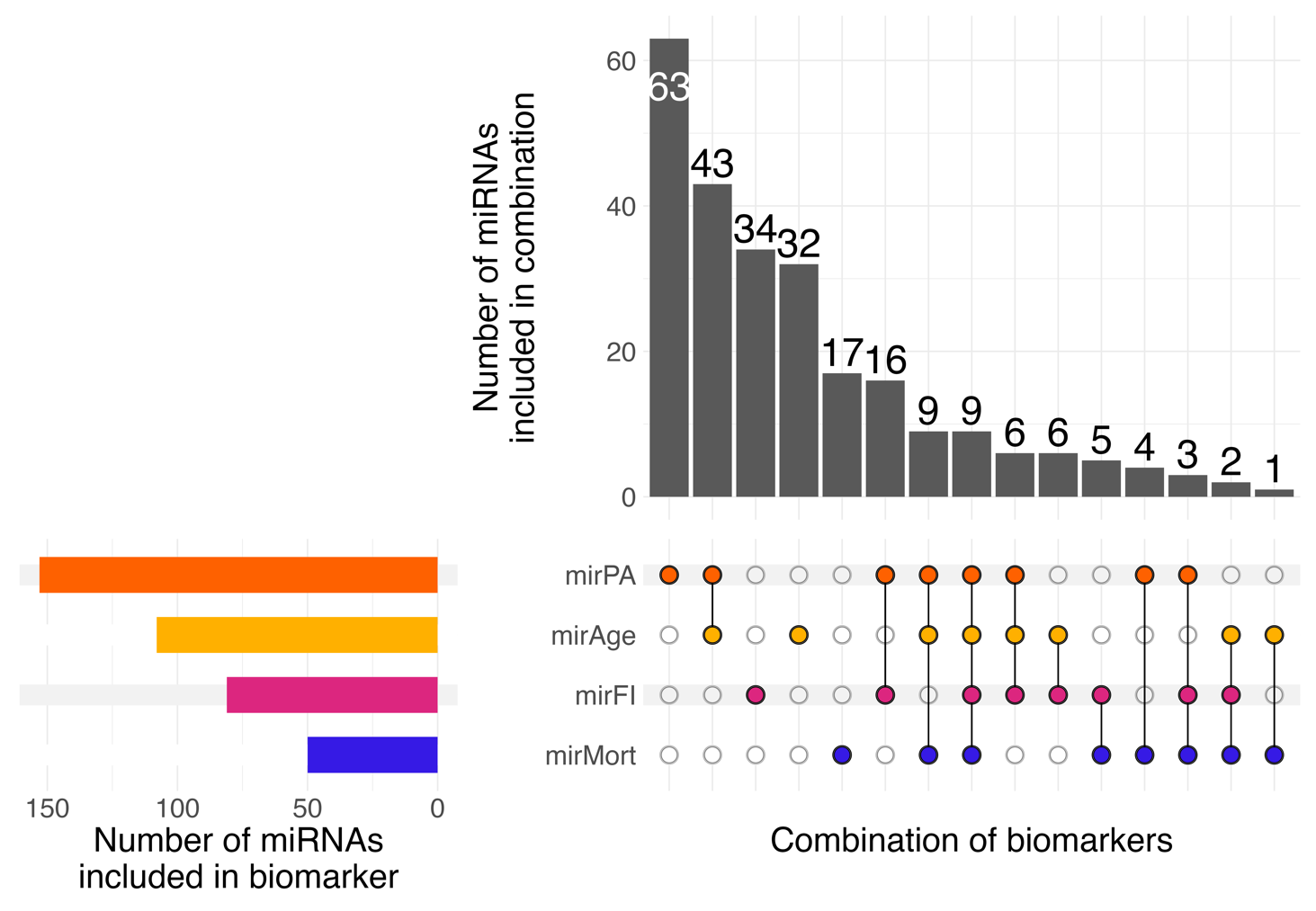
**

Figure represents the overlap between miRNAs selected in the different miRNA-based aging biomakers. The colored bar plots at the left side demonstrate the number of miRNAs selected in each biomarker. The bar plots on top show the number of miRNAs included in the different combinations of biomarkers, the combinations are shown below when a dot is filled the outcome is included in the combination. Only miR-335-5p (mirAge: 0.01076924; mirPA: -0.0596777), only miR-1229-3p (mirAge: 0.08725706; mirFI: -0.001598291), miR-7106-5p (mirAge: 0.15898800; mirFI: -0.000281850), miR-660-5p (mirPA: 0.3383700; mirFI: -0.005818589), and miR-7111-5p (mirFI: -0.001517861; mirMort: 0.0158228) had different directions across the aging biomarkers
