# Supplementary material for: Plasma microRNA signatures of aging and their links to health outcomes and mortality: findings from a population-based cohort study
Source: Genome Med. 2025 Jun 25;17:70. doi: 10.1186/s13073-025-01437-5 (PMC12188677; doi:10.1186/s13073-025-01437-5)
Supplement: Supplementary file 14 — Additional file 14: Figure S8. Spearman’s correlations between miRNA Age and chronological age in the test and validation sets. [file 13073_2025_1437_MOESM14_ESM.docx]

Additional file 14. Figure S8. Spearman’s correlations between miRNA Age and chronological age in the test and validation set.

MirAge indicates miRNA Age; mirPA, miRNA PhenoAge; mirFI, miRNA frailty index (FI); mirMort, miRNA Mortality.

Text in normal font refers to raw miRNA-based aging biomarkers, while text in italics indicates age-accelerated miRNA-based biomarkers. Values represent Spearman’s rank correlation in **a.** the test set and **b.** the validation set; the background color is darker for higher correlations.
