# Supplementary material for: Plasma microRNA signatures of aging and their links to health outcomes and mortality: findings from a population-based cohort study
Source: Genome Med. 2025 Jun 25;17:70. doi: 10.1186/s13073-025-01437-5 (PMC12188677; doi:10.1186/s13073-025-01437-5)
Supplement: Supplementary file 15 — Additional file 15: Table S10. Associations between standardized age-accelerated miRNA aging biomarkers and continuous measure. [file 13073_2025_1437_MOESM15_ESM.docx]

Additional file 15: Table S10. Associations between standardized age-accelerated miRNA aging biomarkers and continuous measures.

|  |  | MiRNA Age | | MiRNA PhenoAge | | MiRNA FI | | MiRNA Mortality | |
| --- | --- | --- | --- | --- | --- | --- | --- | --- | --- |
|  |  | B(CI) | pFDR | B(CI) | pFDR | B(CI) | pFDR | B(CI) | pFDR |
| Test set (*n*=772) | Frailty Index (*n*=757) | 0.04 (-0.03;0.10) | 0.38 | 0.12 (0.06;0.19) | 7.51x10^-4^ | 0.17 (0.11;0.23) | 4.841x10^-7^ | 0.13 (0.07;0.20) | 2.55x10^-4^ |
|  | Delta frailty (*n*=539) | 0.04 (-0.04;0.12) | 0.43 | 0.08 (0.00;0.16) | 0.10 | -0.01 (-0.09;0.07) | 0.91 | 0.04 (-0.04;0.12) | 0.41 |
|  | PhenoAge (*n*=770) | 0.11 (0.07;0.14) | 1.70x10^-7^ | 0.20 (0.16;0.23) | 3.67x10^-25^ | 0.17 (0.13;0.20) | 7.71x10^-20^ | 0.17 (0.13;0.21) | 6.62x10^-19^ |
|  | BADL (*n*=752) | 0.04 (-0.02;0.11) | 0.30 | 0.09 (0.03;0.16) | 0.01 | 0.12 (0.06;0.18) | 3.79x10^-4^ | 0.10 (0.03;0.16) | 7.19x10^-3^ |
|  | IADL (*n*=528) | 0.02 (-0.06;0.10) | 0.68 | 0.07 (0.00;0.15) | 0.09 | 0.06 (-0.01;0.13) | 0.17 | 0.05 (-0.02;0.13) | 0.24 |
| Validation set (*n=*754) | BADL (*n*=752) | 0.08 (0.01;0.14) | 0.06 | 0.10 (0.03;0.17) | 7.66x10^-3^ | 0.14 (0.08;0.21) | 1.04x10^-4^ | 0.12 (0.06;0.19) | 9.24x10^-4^ |
|  | IADL (*n*=588) | 0.04 (-0.04;0.12) | 0.47 | 0.06 (-0.02;0.14) | 0.28 | 0.09 (0.01;0.17) | 0.06 | 0.09 (0.01;0.18) | 0.05 |

B indicates beta coefficient per standard deviation increase; BADL, basic activities of daily living; CI, 95%-confidence interval; IADL, instrumental activities of daily living; n, number of participants; pFDR, p-value after false discovery rate correction.
