# Supplementary material for: Plasma microRNA signatures of aging and their links to health outcomes and mortality: findings from a population-based cohort study
Source: Genome Med. 2025 Jun 25;17:70. doi: 10.1186/s13073-025-01437-5 (PMC12188677; doi:10.1186/s13073-025-01437-5)
Supplement: Supplementary file 16 — Additional file 16: Table S11. Associations of standardized miRNA-based aging biomarkers with self-reported health in the validation set. [file 13073_2025_1437_MOESM16_ESM.docx]

Additional file 16: Table S11. Associations of standardized miRNA-based aging biomarkers with self-reported health in the validation set.

|  | Self-reported better health than peers (n_cases_=299/ n=721) | | Self-reported worse health than peers (n_cases_=119/ n=721) | |
| --- | --- | --- | --- | --- |
|  | OR(CI) | pFDR | OR(CI) | pFDR |
|  | Ref. Self-reported health comparable to peers | | | |
| MiRNA Age | 1.08 (0.90;1.28) | 0.48 | 1.33 (1.06;1.68) | 0.03 |
| MiRNA PhenoAge | 0.91 (0.76;1.09) | 0.41 | 1.47 (1.17;1.84) | 2.49x10^-3^ |
| MiRNA FI | 0.76 (0.64;0.91) | 6.43x10^-3^ | 1.35 (1.08;1.69) | 0.02 |
| MiRNA Mortality | 0.85 (0.71;1.02) | 0.14 | 1.46 (1.17;1.83) | 2.49x10^-3^ |

CI indicates 95%-confidence interval; n_cases_ number of cases; n, number of participants; OR, odds ratios; pFDR, p-value after false discovery rate correction.
