# Supplementary material for: Plasma microRNA signatures of aging and their links to health outcomes and mortality: findings from a population-based cohort study
Source: Genome Med. 2025 Jun 25;17:70. doi: 10.1186/s13073-025-01437-5 (PMC12188677; doi:10.1186/s13073-025-01437-5)
Supplement: Supplementary file 17 — Additional file 17: Table S12. Associations between standardized miRNA-based aging biomarkers and first morbidity, multi-morbidity, and mortality. [file 13073_2025_1437_MOESM17_ESM.docx]

Additional file 17: Table S12. Associations between standardized miRNA-based aging biomarkers and first morbidity, multi-morbidity, and mortality.

|  | Endpoint  (n_cases_/n) | MiRNA Age | | MiRNA PhenoAge | | MiRNA FI | | MiRNA Mortality | |
| --- | --- | --- | --- | --- | --- | --- | --- | --- | --- |
|  |  | HR(CI) | pFDR | HR(CI) | pFDR | HR(CI) | pFDR | HR(CI) | pFDR |
| Test set (*n*=772) | All-cause mortality (434/770) | 1.15 (1.06;1.24) | 2.20x10^-3^ | 1.23 (1.13;1.34) | 1.09x10^-5^ | 1.21 (1.11;1.33) | 1.92x10^-4^ | 1.22 (1.12;1.33) | 3.90x10^-5^ |
|  | First morbidity (223/491) | 0.86 (0.75;0.98) | 0.04 | 0.92 (0.82;1.04) | 0.28 | 1.05 (0.93;1.20) | 0.48 | 0.95 (0.84;1.07) | 0.44 |
|  | Multi-morbidity (97/725) | 0.96 (0.81;1.13) | 0.68 | 1.00 (0.86;1.16) | 0.97 | 1.09 (0.93;1.28) | 0.37 | 0.99 (0.85;1.16) | 0.96 |
| Validation set (*n*=754) | All-cause mortality (45/747) | 1.06 (0.77;1.46) | 0.77 | 1.12 (0.87;1.45) | 0.44 | 1.16 (0.88;1.52) | 0.39 | 1.02 (0.77;1.33) | 0.94 |

CI indicates 95%-confidence interval; HR, hazard ratio per standard deviation increase; n_cases_ number of cases; n, number of participants; pFDR, p-value after false discovery rate correction.
