# Supplementary material for: Plasma microRNA signatures of aging and their links to health outcomes and mortality: findings from a population-based cohort study
Source: Genome Med. 2025 Jun 25;17:70. doi: 10.1186/s13073-025-01437-5 (PMC12188677; doi:10.1186/s13073-025-01437-5)
Supplement: Supplementary file 18 — Additional file 18: Table S13. Associations between standardized miRNA-based aging biomarkers and all-cause mortality with and without adjustment for PhenoAge and the frailty index. [file 13073_2025_1437_MOESM18_ESM.docx]

Additional file 18: Table S13. Associations between standardized miRNA-based aging biomarkers and all-cause mortality with and without adjustment for PhenoAge and the frailty index.

|  | M1: Adjusted for sex, cell counts, being on the border, and plate number | | | M1 + Additional adjustment for baseline PhenoAge | | | M1 + Additional adjustment for baseline frailty index | | |
| --- | --- | --- | --- | --- | --- | --- | --- | --- | --- |
|  | N_cases_/N | HR(CI) | pFDR | N_cases_/N | HR(CI) | pFDR | N_cases_/N | HR(CI) | pFDR |
| MiRNA Age | 472/769 | 1.15 (1.06;1.24) | 2.20x10^-3^ | 472/769 | 1.16 (1.07;1.26) | 7.97x10^-4^ | 464/756 | 1.15 (1.06;1.24) | 1.67x10^-3^ |
| MiRNA PhenoAge | 472/769 | 1.23 (1.13;1.34) | 1.09x10^-5^ | 472/769 | 1.29 (1.18;1.41) | 2.26x10^-7^ | 464/756 | 1.23 (1.12;1.34) | 2.45x10^-5^ |
| MiRNA FI | 472/769 | 1.21 (1.11;1.33) | 1.92x10^-4^ | 472/769 | 1.24 (1.13;1.36) | 3.33x10^-5^ | 464/756 | 1.20 (1.09;1.32) | 4.26x10^-4^ |
| MiRNA Mortality | 472/769 | 1.22 (1.12;1.33) | 3.90x10^-5^ | 472/769 | 1.27 (1.16;1.39) | 3.16x10^-6^ | 464/756 | 1.21 (1.11;1.32) | 1.03x10^-4^ |

CI indicates 95%-confidence interval; HR, hazard ratio per standard deviation increase; n_cases_ number of cases; n, number of participants; pFDR, p-value after false discovery rate correction.
